# Chemical Identification and Antioxidant Screening of Bufei Yishen Formula using an Offline DPPH Ultrahigh-Performance Liquid Chromatography Q-Extractive Orbitrap MS/MS

**DOI:** 10.1155/2022/1423801

**Published:** 2022-10-15

**Authors:** Jinyan Wu, Bangrong Cai, Ang Zhang, Peng Zhao, Yan Du, Xuefang Liu, Di Zhao, Liu Yang, Xinguang Liu, Jiansheng Li

**Affiliations:** ^1^Collaborative Innovation Center for Chinese Medicine and Respiratory Diseases Co-constructed by Henan Province & Education Ministry of China, Academy of Chinese Medical Sciences, Henan University of Chinese Medicine, Zhengzhou, China; ^2^Henan Key Laboratory of Chinese Medicine for Respiratory Disease, Henan University of Chinese Medicine, Zhengzhou, China; ^3^The First Affiliated Hospital of Henan University of Chinese Medicine, Zhengzhou, China

## Abstract

Chronic obstructive pulmonary disease (COPD) has high morbidity and mortality and presents a threat to human health worldwide. Numerous clinical trials have confirmed that Bufei Yishen formula (BYF), an herbal medicine, can alleviate the symptoms of COPD by reducing oxidative stress-mediated inflammation. However, the active components of BYF remain unclear. We developed an efficient ultrahigh-performance liquid chromatography Q-Extractive Orbitrap mass spectrometry method to identify the composition of BYF and determine its antioxidant profile through an offline screening strategy based on 1,1-diphenyl-2-trinitrophenylhydrazine (DPPH)-liquid chromatography-mass spectrometry. In total, 189 compounds were identified in BYF extract, including 83 flavonoids, 24 lignans, 20 alkaloids, 15 saponins, 11 terpenoid, 10 saccharides, eight lipids, seven organic acids, two coumarins, two amino acids, and seven other compounds. Among them, 79 compounds were found to have a potential antioxidant activity. *In vitro* validation indicated that the free radical scavenging activities of rosmarinic acid and calycosin were similar to that of the positive control (DPPH IC_50_ = 25.72 ± 1.02 and 147.23 ± 25.12 *μ*g/mL, respectively). Furthermore, calycosin had a high content in serum after the oral administration of BYF, indicating that calycosin might be the major antioxidant compound in BYF.

## 1. Introduction

Chronic obstructive pulmonary disease (COPD) is a major chronic disease with high morbidity and mortality, especially among the elderly and smokers [1]. According to the World Health Authority and Global Initiative for Chronic Obstructive Lung Disease, bronchodilators and corticosteroids along with nonpharmacologic therapies such as pulmonary rehabilitation are frequently used to treat COPD. Although these therapies can reduce exacerbations and alleviate symptoms, there is little evidence to suggest that they can suppress the progression of COPD. Various recent clinical trials have suggested that herbal medicines have the potential to improve symptoms, reduce the frequency of acute exacerbation, and improve the quality of life for COPD patients [1]. Bufei Yishen formula (BYF) is an oral prescription for COPD that has proven clinically effective for COPD control [2]. The use of BYF and BYF combined with other therapies (e.g., acupoint sticking, electroacupuncture, and Tongsai granules) have shown beneficial effects in terms of lung function, clinical symptoms, quality of life, and acute exacerbation frequency in patients with stable COPD [3–5]. In a COPD rat model exposed to cigarette smoke and bacteria, BYF also ameliorated airway inflammation and remodeling [6–11]. We previously demonstrated that the mechanism of BYF against COPD might involve reducing inflammatory cytokines and oxidative stress, regulating immune response and lipid metabolism [12–15], restoring the Th17/Treg balance by activating adenosine 2a receptor [16], modulating the activities of STAT3 and STAT5 in COPD rats [17], and suppressing interleukin expression and/or secretion [18]. However, the effective substances of BYF are not clear at present, which forms a bottleneck problem in the further development of the preparation. It is well known that the ingredients of traditional Chinese medicine are complex, the unclear of effective substances make it difficult to select the biomarkers for quality control.

Oxidative stress triggering sustained inflammatory response is a major contributing factor in COPD [19, 20]. A system analysis integrating transcriptomics, proteomics, and metabolomics showed that the target proteins of BYF against COPD are glutamate-cysteine ligase, glutathione reductase, G6PD, glutathione S-transferase P, glutathione S-transferase A1/2, GSTM1/2, and SOD1, which are predominantly enriched in oxidative stress-related pathways [15]. We speculated that the antioxidant profiling of BYF might help uncover the effective substances of BYF.

**Figure 1:**
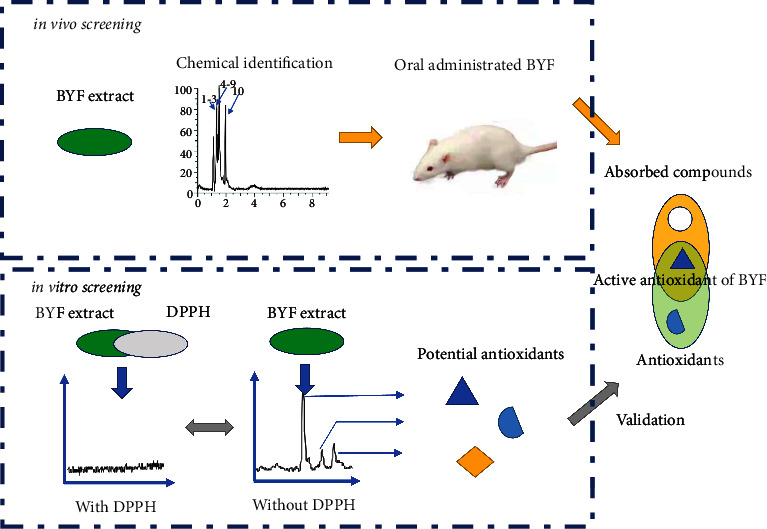
Schematic diagram of antioxidant screening using the offline DPPH-UHPLC-Q-extractive-Orbitrap-MS/MS system.

At present, “separation-activity verification” is the main strategy for screening antioxidant substances in herbal medicines. In this approach, as many natural products as possible are isolated from the herbal medicine, and their activities are evaluated through antioxidant assays. However, the separation step in this method is time-consuming. Ultrahigh-performance liquid chromatography (UHPLC) coupled with high-resolution mass spectrometry (HRMS) has shown great potential for the rapid identification of antioxidants in natural products [21–23]. It is based on the hypothesis that the reaction of antioxidants with 1,1-diphenyl-2-trinitrophenylhydrazine (DPPH) will significantly reduce the concentrations of compounds with potential antioxidant activity. Due to the accurate mass measurement provided by UHPLC-HRMS, the antioxidants in herbal medicines can be easily screened and identified. In this work, an efficient UHPLC Q-Extractive Orbitrap MS/MS method was developed to elucidate the chemical composition of BYF. The antioxidants were identified by offline DPPH-UHPLC Q-Extractive Orbitrap MS/MS and their concentrations were detected in rat serum after the oral administration of BYF.  Figure 1 shows a schematic diagram of the experimental design. This study combines rapid antioxidant screening based on the chromatography-activity relationship with an evaluation of drug absorption to indicate the antioxidant substances contained in BYF. This allows to quickly identify the antioxidants that really effective *in vivo*, and the proposed strategy also provides reference for the screening of antioxidants of other traditional Chinese medicines.

## 2. Experimental Methods

### 2.1. Reagents and Materials

Methanol (HPLC-grade), acetonitrile (LC-MS grade), and formic acid were purchased from Thermo Fisher Technology Co., Ltd. (Shanghai, China). Ethanol was purchased from Mreda Technology Inc. (Beijing, China). Ultrapure water was produced by a laboratory Milli-Q system (Merck Millipore, Shanghai, China). DPPH and potassium persulfate were purchased from Shanghai Macklin Biochemical Chemical Co. Ltd. (Shanghai, China). The fresh DPPH radical solution was kept away from light. 2,2-Azino-bis-(3-ethylbenzothiazoline-6-sulfonic acid) diammonium salt (ABTS) and L-ascorbic acid were purchased from Sigma-Aldrich Co. (St. Louis, MO, USA). All standard references included in Table 1 were purchased from Shanghai Yuanye Technology Co., Ltd. (Shanghai, China).

### 2.2. Animals

Adult Sprague-Dawley (SD) male SPF rats weighing 170–200 g were provided by Beijing Weitong Lihua Experimental Animal Company (animal license number SCXK (Yu) 2020-0004). All animals were fed standard feed and were allowed to drink freely for one week at 20°C–25°C. On the day before the experiment, the animals were fasted for 12 h (except for drinking water). All protocols were approved by the Ethics Committee of the Henan University of Chinese Medicine (approval number DWLLGZR202202029).

### 2.3. Sample Preparation

#### 2.3.1. Preparation of BYF

All medicinal herbs in BYF were obtained from Zhengzhou Ruilong Pharmaceutical Co. (Zhengzhou, China) BYF consists of 12 medicinal materials: *Astragali Radix* (AR), *Fritillariae thunbergii* Bulbus (FTB), *Pheretima* (P), *Citri reticulatae Pericarpium* (CRP), *Ardisia japonicae Herba* (AJH), *Epimedii folium* (EF), *Ginseng radix et rhizoma* (GRR), *Schisandrae chinensis* Fructus (SCF), *Lycii fructus* (LF), *Perillae fructus* (PF), *Corni fructus* (CF), and *Paeoniae radix* Rubra (PRR). Extraction of AR, LF, EF, PRR, and P were conducted by 12 L of water per gram of crude materials by two times. After filtering, the filtrate was concentrated. Reflux extraction of GRR, FTB, CRP, AJH, SCF, PF, and CF were conducted by 10 L of 70% ethanol per gram of crude materials by 2 times. After filtering, the ethanol was recovered from the filtrate and combined with the abovementioned concentrate. The combined concentrate was further concentrated into a thick paste with a relative density of 1.18–1.22 (60°C). Finally, the per-gram dry extract obtained was equivalent to 3.81 g of raw medicinal herb.

BYF extract (0.2 mg) was extracted by ultrasonication with 20 mL of 70% methanol for 30 min. The extract was filtered, and the filtrate was centrifuged at 13000 rpm for 10 min at 4°C. The supernatant was stored at −20°C before analysis. All reference standards and internal standards (IS) were dissolved in methanol at concentrations of 10 *μ*g/mL.

For antioxidant capacity assay, BYF extract, different concentrations of L-ascorbic acid as the positive control (0.5–100 *μ*g/mL) and candidate antioxidants (0.5–1000 *μ*g/mL) were prepared in methanol.

For antioxidant quantification, stock solutions of the standard references and IS were prepared in methanol at concentrations ranging from 0.515 to 1.23 mg/mL. A series of working solutions of mixed reference standards were obtained by further dilution with methanol. Calibration standards were prepared by spiking 10 *μ*L of the standard solutions into 90 *μ*L of blank biological samples to obtain final concentrations of 0.0124–1080 ng/mL. All working solutions were stored at −80°C. Calibration curves were acquired by plotting the peak area ratio (*y*) of each compound to the IS against the corresponding concentration of each compound (*x*). The acceptance criterion of a calibration curve was a correlation coefficient (*r*) of 0.99 or better along with relative errors for each point within ±15%.

#### 2.3.2. Preparation of Serum Samples

The rats were randomly divided into the normal group and BYF group, with six rats in each group. The rats in the BYF group were gavaged twice per day with 18.28 g/kg/d BYF for 7 d. The normal group was given the same amount of normal saline by gavage for 7 d. The rats were fasted for 1 d before blood collection. Blood was collected from the orbital vein at 10 min, 30 min, 1 h, 2 h, and 4 h after the last gavage, and centrifuged at 3000 rpm for 10 min at 4°C. Finally, the serum was separated and stored at −80°C for later use.

To qualitative analyze the BYF components in rat serum after oral administration, we randomly mixed five serum samples from each group collected at different times after administration (20 *μ*L for each sample, 100 *μ*L in total). For the quantitative analysis of BYF components in rat serum, 100 *μ*L of the serum samples collected at different times after BYF administration was taken and analyzed.

A protein precipitation procedure was used to extract BYF components. An 100-*μ*L aliquot of serum was spiked with 300 *μ*L of acetonitrile (containing 200 ng/mL IS and 6 ng/mL ascorbic acid). The mixture was vortex-mixed for 3 min, allowed to stand at 4°C for 5 min, and centrifuged at 4°C and 18534 g for 10 min. Next, 300 *μ*L of the supernatant was transferred into a new tube and evaporated to dryness using an Integrate SpeedVac System (ThermoFisher Scientific Corporation, USA). The residue was redissolved in 50 *μ*L of 50% acetonitrile, and a 35-*μ*L aliquot of the supernatant was collected for analysis.

### 2.4. Chromatography and MS Conditions

The samples were analyzed by LC-MS/MS using a Dionex Ultimate 3000 UPLC system (ThermoFisher Scientific, Germering, Germany) coupled to a Thermo Scientific Q-Exactive Orbitrap mass spectrometer (ThermoFisher Scientific, Bremen, Germany).

To identify the BYF components in the extract and serum, the samples were loaded onto a Phenomenex Synergi Polar-RP column (2 × 150 mm, 4 *μ*m) at 40°C. Mobile phase A was composed of water and 0.1% formic acid. Mobile phase B was composed of ACN and 0.1% formic acid. The flow rate was 0.3 mL/min. The gradient elution conditions were as follows: 0% B (0–5 min); linear gradient from 0% B to 5% B (5–7 min); 5% B to 20% B (7–10 min); 20% B to 25% B (10–20 min); 25% B to 50% B (20–23 min); 50% B to 100% B (23–40 min); 100% B for 3 min (40–43 min); back to 0% B over 2 minutes; 0% B for 5 min (45–50 min). The injection volume was 5.00 *μ*L.

For the quantitative analysis of antioxidants in rat serum, the samples were loaded onto an Agilent ZORBAX-Extend-C18 LC column (4.6 × 50 mm, 1.8 *μ*m) at 30°C. Mobile phase A was composed of water and 0.1% formic acid. Mobile phase B was composed of acetonitrile and 0.1% formic acid. The flow rate was 0.5 mL/min. The gradient elution condition were as follows: 5% B (0–2 min); linear gradient from 5% B to 23% B (2-3 min); 23% B for 6 min (3–9 min); 23% B to 50% B (9-10 min); 50% B to 68% B (10–15 min); 68% B for 3 min (15–18 min); 68% B to 100% B (18–22 min); 100% B for 2 min (22–24 min); back to 5% B (24-25 min); and 5% B for 2 min (25–27 min). The injection volume was 5 *μ*L.

The mass spectrometer was equipped with a heated electrospray ionization probe. The spray voltage was set at 3500 V for positive ion mode and 2800 V for negative ion mode. The flow rates of the sheath gas and aux gas were 40 and 10 Arb, respectively. The capillary temperature was 325°C, and the aux gas heater temperature was 300°C. Full scans from *m/z* 100 to 1500 were performed in the Orbitrap at a resolution of 70 K for quantification. The AGC target value was 3 × 10^6^, and the maximum injection time was 200 ms. Parallel reaction monitoring (PRM) mode was used for fragmentation identification and quantification of BYF metabolites. The target MS2 scan in PRM mode was conducted at a resolution of 17.5 K with an isolation width of 4.0 Da, an AGC target value of 2 × 10^5^, and a maximum injection time of 100 ms. The precursor ion/product ions and normalized collision energy for each compound are listed in Table S1 [29].

### 2.5. Antioxidant Profiling

#### 2.5.1. Offline DPPH-UHPLC Q-Extractive Orbitrap MS/MS

BYF extract (100 *µ*L) was mixed with DPPH solutions of different concentrations (100 *µ*L and 0.5, 1, 2, 5, and 10 mM), and the mixtures were incubated in the dark at room temperature for 30 min. The mixtures were further monitored by UHPLC-Q-Extractive Orbitrap MS/MS. Control experiments in which DPPH solution was replaced by a blank solution were carried out for comparison. The reduction in the peak area compared with the control group indicated the DPPH radical scavenging activity of the compounds in BYF.

#### 2.5.2. Determination of Antioxidant Activities

In order to determine the antioxidant activity of potential antioxidants, DPPH radical scavenging assay, ABTS radical scavenging activity, and ferric-reducing antioxidant power (FRAP) assay were conducted.

*(1) DPPH radical scavenging assay*. The DPPH radical scavenging assay was performed on a spectrophotometer microplate reader from ThermoFisher Scientific (Vantaa Finland) using multiwell plates as a previously published method described [30]. The DPPH solution was diluted by methanol to 0.1 mM as a working solution. The reaction was initiated by mixing 50 *μ*L of test solution with 150 *μ*L of DPPH working solution and incubated in dark at room temperature for 30 min. Monitoring of the absorbance at 517 nm was carried out after the reaction was completed. The scavenging capacity of samples were calculated by experimental scavenging capacity (ESC) using equation (1) as follows:

(1) $$\begin{array}{c} \%\text{ ESC}=100-\left\{\frac{\left[\left({\text{Abs}}_{\text{sample}}-{\text{Abs}}_{\text{blank}}\right)\times 100\right]}{{\text{Abs}}_{\text{control}}}\right\}, \end{array}$$

where Abs_sample_ is the absorbance value of the sample (DPPH solution plus antioxidant) at each time interval and Abs_blank_ is the absorbance value of the blank (methanol plus antioxidant(s)). Abs_control_ is the absorbance value of control (methanol plus DPPH solution).

The value of 50% inhibition (IC_50_) was calculated by the graph plotting sample concentration and inhibition percentage.

*(2) ABTS radical scavenging activity*. The ABTS radical scavenging activity of the crude extracts was determined using the method described by Zhou et al. [30] with minor modifications. Aqueous ABTS (7 mM) was mixed with 2.45 mM aqueous potassium persulfate (1 : 1, *v*/*v*), and the solution was left to react for 16 h at room temperature in the dark. The ABTS•+ solution was diluted with absolute ethanol to an absorbance at 734 nm of 0.70 ± 0.02 to obtain an ABTS•+ radical working solution. Then, 160 *μ*L of the ABTS•+ radical working solution was mixed with 40 *μ*L of test solutions, and the mixture was incubated for 6 min. The absorbance of the mixture was measured at 734 nm. The ABTS radical scavenging assay was performed on a spectrophotometer microplate reader. The ABTS radical scavenging activity was calculated according to the following equation:

(2) $$\begin{array}{c} \text{ABTS radical scavenging activity }\%=\left[\frac{\left(A\text{ blank}-A\text{ sample}\right)}{A\text{ blank}}\right]\times 100, \end{array}$$

where *A* sample = the absorbance at 734 nm with sample and *A* blank = the absorbance at 734 nm without sample. The IC_50_ value was calculated and represents the concentration necessary to reduce the maximum response of the ABTS by half.

*(3) FRAP assay*. The FRAP assays were performed by a total antioxidant capacity assay kit with the PRAP method according to manufacturer's instruction (Beyotime Biotech Inc, Shanghai, China). Briefly, 180 *μ*L FRAP working solution was mixed with 5 *μ*L extract of BYF, or 5 *μ*L distilled water as blank control, or 5 *μ*L 0.15–1.5 mM FeSO_4_ standard solution (dissolved in distilled water) as standard curve. The absorbance of the mixture was measured at 593 nm after incubation at 37°C for 3–5 minutes. The total antioxidant capacity of the sample was calculated according to the standard curve. For FRAP method, the total antioxidant capacity of the extract is expressed by the concentration of FeSO_4_ standard solution with equivalent antioxidant capacity.

## 3. Results and Discussion

### 3.1. Chemical Identification of BYF Components

**Figure 2:**
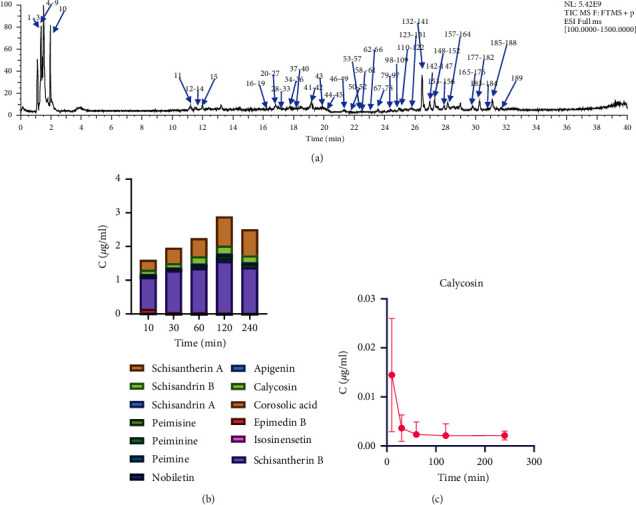
(a) Total ion chromatograms of BYF obtained in positive ion mode. (b) Serum concentrations of 13 components of BYF after oral administration. (c) Mean serum concentration–time profile of calycosin after the oral administration of BYF.

We developed an UHPLC-Q-Extractive Orbitrap-MS/MS method for the comprehensive characterization of the chemical constituents of BYF extract. The total ion chromatography obtained in positive ion mode is shown in Figure 2(a). First, by consulting literature and the Encyclopedia of Traditional Chinese Medicine, we constructed a MS information database of the components of the materials in BYF. In this library, CRP are the dried pericarps of the ripe fruits of *Citrus reticulate* Blanco or its cultivars. CRP mainly contain flavonoids, by UHPLC-QTOF MS, Duan et al. identified 75 flavonoids from CRP [24]. PRR are the roots of *Paeonia lactiflora* and *Paeonia anomala* subsp. Veitchii, which mainly contain monoterpene glycosides, flavonoids, tannins, phenols and paeonols [25]. GRR are the dry roots and rhizomes of *Panax ginseng* C. A. Mey. GRR mainly contain triterpene saponins, which are also widely recognized as active components. Qi et al. identified 70 saponins from GRR [27]. PF are the dry ripe fruits of *Perilla frutescens* (L.) Britt., which mainly contain phenolic acids, triterpenoids, flavonoids and fatty acids [28]. AR are the dry root of *Astragalus membranaceus* (Fisch.) Bge.var.mongholicus (Bge.) Hsiao or *Astragalus membranaceus* (Fisch.) Bge., which mainly contain triterpene saponins and flavonoids, Chu et al. identified 22 astragalosides from AR [26], and Mei et al. totally identified 47 saponins and 55 flavonoids [31]. FTB are the dry bulb of *Fritillaria cirrhosa* D. Don, *Fritilaria unibracteata* Hsiao et K. C. Hsia, *Fritillaria przewalskii* Maxim., *Fritillaria delavayi* Franch., *Fritillaria taipaiensis* P. Y. Li, or *Fritillaria unibracteata* Hsiao et K. C. Hsiavar. wabuensis (S. Y. Tanget S. C. Yue) Z. D. Liu, S. Wang et S. C. Chen, alkaloids are the main components in FTB, terpenoids and steroids can also be found in FTB [32]. *P* are the dry body of *Pheretima aspergillum*, *Pheretima vulgaris* Chen, *Pheretima guillelmi* or *Pheretima pectinifera* Michaelsen, its main components are amino acids and organic acids. Zhang et al. identified 11 free amino acid, 26 organic acids, 11 nucleosides, 5 dipeptides and cyclic dipeptides, and 21 nitrogenous substances from *P* [33]. AJH are the dry whole herb of *Ardisia japonica* (Thunb.) Blume. The main components in AJH including benzoquinones, phenols, flavonoids, chromones, triterpenes, and triterpene saponins [34]. EF are the dry leave of *Epimedium brevicornu* Maxim., *Epimedium sagittatum* (Sieb. et Zucc.) Maxim., *Epimedium pubescens* Maxim. or *Epimedium koreanum* Nakai. EF mainly contain flavonoids, in addition, lignans, polysaccharides and alkaloids can also be detected [35, 36]. SCF are the dry ripe fruit of *Schisandra chinensis* (Turcz.) Baill., SCF mainly contain lignans, and also polysaccharide volatile oil [37]. LF are the fruit of *Lycium barbarum* L., mainly contain polysaccharides, peptide, alkaloids, flavonoids, terpenes, organic acids, lignans, phenolic amides and carotenoids [38]. CF are the dry ripe sarcocarp of *Cornus officinalis* Sieb. et Zucc (Cornaceae), include mainly irridoids, organic acids, triterpenes, cornustannins, and carbohydrates [39].

As shown in Table 1, 189 chemical constituents were identified in BYF based on the library; their MS/MS spectra were matched with online databases and/or published references. Structurally, the main components of BYF were flavonoids (83 compounds), lignans (24 compounds), and alkaloids (20 compounds). Other identified components included 15 saponins, 11 terpenoids, 10 saccharides, eight lipids, seven organic acids, two coumarins, two amino acids, and seven other compounds. Among the identified compounds, 37 were identified by comparison with the retention times and MS spectra of standards; the MS/MS spectra of these compounds are shown in Supplementary Materials Figures S1–S37.

#### 3.1.1. Flavonoids

**Figure 3:**
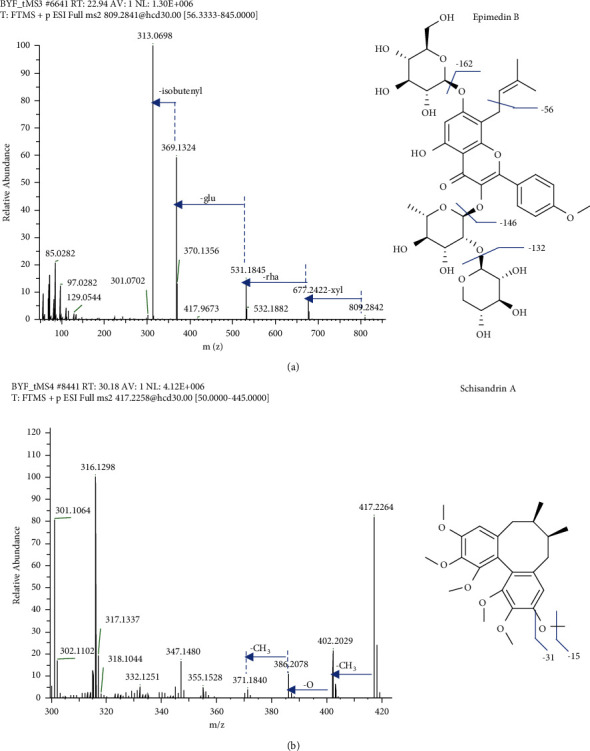
MS/MS spectra and structure of epimedin B (a) and schisandrin A (b).

A total of 83 flavonoids were identified in BYF. Among them, 33 flavonoids were from only EF. These flavonoids are mainly flavonoids with isobutenyl at the C-8 position and their glycosides including icariin, epimedin A, and compounds 17, 54, 55, 60, 61, 63, 68, 70, 76, 77, 81, 83, 87, 93, 98, 99, 106, 110, 111, 113, 114, 120, 122, 127, 132, 133, and 166. These flavonoids have the characteristic isobutenyl neutral loss of 56 Da as a diagnostic ion (Table 1). For flavonoid glycosides, the common neutral losses of 162 and 146 Da were due to the presence of glucosyl and rhamnosyl groups. For example, in Figure 3(a), Epimedin B at the retention time of 22.98 min had a positively charged molecular ion ([M + H]^+^) at *m/z* 809.2841, which yielded secondary fragments at *m/z* 677.2428 ([M + H-xyl]^+^), 531.1852 ([M + H-xyl-rha]^+^), 369.1324 ([M + H-xyl-rha-glu]^+^), and 313.0698 ([M + H-xyl-rha-glu-isobutenyl]^+^). In addition, 31 flavonoids (mainly flavonoid aglycones) were derived from CRP. The summary of the MS/MS fragments of CRR flavonoids reported by Duan et al. [24] was used for the structural identification of flavonoid aglycones in this work, especially those whose structures were not completely determined. The other flavonoids were mainly from FTB, GRR, LF, PF, and AR.

#### 3.1.2. Lignans

A total of 24 lignans were identified in BYF, all of which were from SCF. More than 150 lignans were isolated from SCF, mainly biphenyl cyclooctadienes, spirobenzofuran biphenyl cyclooctadienes, 4-aryltetrahydronaphthalene, 2,3-dimethyl-1,4-diarylbutane, and 2,5-diaryltetrahydrofurans. Among them, biphenyl cyclooctadienes have the most species and the strongest biological activity [40]. Biphenyl cyclooctadienes include schisantherin A, B, and C, gomisin L1, and schisandrin A, B, and C. The characteristic neutral losses of C_4_H_6_COOH, CH_3_OH, CO_2_, CO, CH_3_, and H_2_O were attributed to the presence of 2-methylbutyryl, hydroxymethyl, carboxyl, carbonyl, methyl, and hydroxyl groups in their structures (Table 1). For example, in Figure 3(b), Schisandrin A at the retention time of 30.27 min has a positively charged molecular ion ([M + H]^+^) at *m/z* 809.2841, which yielded secondary fragments at *m/z* 402.2029 ([M + H − CH_3_]^+^), 386.2079 ([M + H − CH_3_ − O]^+^), and 371.1832 ([M + H − 2CH_3_ − O]^+^).

#### 3.1.3. Alkaloids

A total of 20 alkaloids were identified in BYF. Among them, 18 alkaloids were from only FTB. FTB mainly contains steroidal alkaloids such as peimisine and peimine [41]. There are few characteristic fragments of steroidal alkaloids, in which only neutral loss of H_2_O can be found. Therefore, these structures are confirmed by comparing the retention time with the standard. FTB also contains some alkaloid glycosides such as sibelicin glycoside and yibeinoside A. The common neutral loss of 162 Da was attributed to the presence of a glucosyl group (Table 1).

### 3.2. Screening of Antioxidant Components Using the Offline DPPH-UHPLC Q-Extractive Orbitrap MS/MS

As mentioned above, BYF can significantly alleviate the symptoms of COPD in clinical practice. We have also done some research on the mechanism of BYF in treating COPD, the most important is BYF treatment could effectively inhibit the inflammatory response of the lungs [12, 13]. In COPD rats, BYF significantly inhibited the expression of IL-1*β*, IL-6, TNF-*α*, and sTNFR2 induced by cigarette smoke and bacterial infection exposures. The inhibition of BYF on inflammatory response in rat COPD model may be through restoring the Th17/Treg balance by activating adenosine 2a receptor [16] and modulating the activities of STAT3 and STAT5 [17]. Th17/Treg imbalance is considered to be important of COPD development. In COPD patients, the Th17/Treg cell balance shifts toward Th17 cells, which triggers inflammatory responses in the airways and lungs and exacerbates alveolar destruction by producing interleukin-17 [17]. On the one hand, regulating oxidative stress is also an important mechanism of BYF regulating inflammation. We used transcriptomics and proteomics finding that the target proteins of BYF against COPD are enriched in oxidative stress-related pathways [15], These will further inhibit the inflammatory response related to oxidative stress. Therefore, we studied the antioxidant activity of BYF and its antioxidants in order to reveal its effective substances.

#### 3.2.1. Total Antioxidant Capacity of BYF

We evaluated the total antioxidant capacity of BYF by DPPH, ABTS, and FRAP assays.  Table 2 shows that the IC_50_ values of BYF in the DPPH and ABTS assays were 1136.36 ± 148.03 and 602.35 ± 81.26 *μ*g/mL, respectively. In addition, BYF showed high total antioxidant capacity of FRAP (0.51 ± 0.04 mM). Thus, it is necessary to further screen the active components of BYF.

#### 3.2.2. Antioxidant Screening of BYF

DPPH is a stable free radical with an odd electron. DPPH is commonly used to assess the radical scavenging activity of antioxidants; it is capable of accepting one or more hydrogen atoms from an antioxidant, resulting in an unconjugated structure with reduced MS response, which can be detected by HRMS [42, 43]. Moreover, the use of DPPH saves time and labor compared to other free radicals such as ABTS [44]. This antioxidant screening strategy based on the change in MS signal can be divided into online and offline modes. Online screening requires two HPLC pumps, one for chromatographic separation and the other for delivering DPPH solution. The chromatographic fraction and DPPH react online in the pipeline. This method is rapid but has relatively poor stability [44]. Therefore, the more stable and sensitive offline mode was used in this work. In offline mode, the herbal medicine extract was fully reacted with DPPH, and the reaction solution was injected into the mass spectrometer for antioxidant detection.

**Figure 4:**
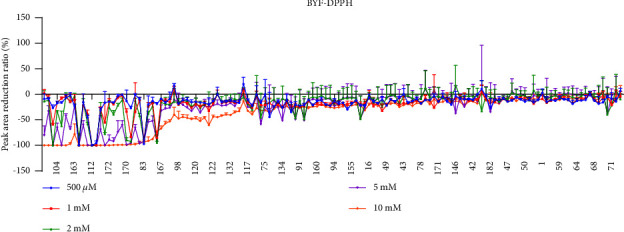
MS Intensity reduction for compounds in BYF after reaction with DPPH at different concentrations (500 *μ*M, 1 mM, 2 mM, 5 mM, and 10 mM). The compound numbers marked on the *X*-axis refer to Table 1.

In an offline experiment, the concentration ratio of DPPH in the extract will significantly affect the efficiency of antioxidant screening [45]. A relative excess of DPPH will not affect the free radical scavenging ability of the active components. However, when the DPPH concentration is insufficient, the free radical scavenging ability cannot be detected [46]. We optimized the DPPH concentration (Figure 4) and found that 10 mM DPPH was most suitable to screen the free antioxidant components. The components with peak intensity decreased more than 20% were considered as potential antioxidants, which are summarized in Table 3.

#### 3.2.3. Antioxidant Activities of the Potential Antioxidants

To verify the antioxidant activities of the potential antioxidants determined above, we measured the free radical scavenging ability of 13 potential antioxidants with available reference standards by DPPH and ABTS assay. As shown in Table 2, 4 compounds showed high free radical scavenging ability for DPPH and or ABTS. Among them, rosmarinic acid had a strongest scavenging activity in DPPH assay (IC_50_ = 25.72 ± 1.02 *μ*g/mL), and rosmarinic acid and calycosin both showed strong scavenging activity in ABTS assay (IC_50_ = 19.00 ± 0.75 and 19.34 ± 5.05 *μ*g/mL, respectively) which superior to ascorbic acid. The results show that phenolic acids and flavonoids in BYF play a major role in the antioxidative activity. Rosmarinic acid has been reported to alleviate oxidative lung damage and airway inflammation based on its strong antioxidant activity [47–49]. Rosmarinic acid can also decrease the population of inflammatory cells; reduce the levels of proinflammatory cytokines such as IL-4, IL-5, and IL-13; upregulate IFN-*γ* secretion; upregulate the activities of SOD, GPx, and CAT; increase Cu/Zn SOD; and significantly downregulate ROS production and the expressions of NOX-2 and NOX-4 in lung tissues [48]. Calycosin has also shown good antioxidant activity [50] and can ameliorate various lung injuries including sepsis, cecal ligation, and puncture by regulating oxidative stress-mediated inflammation in vivo and augmenting superoxide dismutase and glutathione [51]. Since oxidative stress and inflammation are the main pathogeneses of COPD, we suspect that these components are important antioxidants in BYF for the treatment of COPD.

### 3.3. Analysis of Antioxidants in Rat Serum

The antioxidants in BYF may not show the expected antioxidant activity *in vivo* because of their poor absorption after oral administration. To investigate whether the potential antioxidants might be present *in vivo*, rats were orally administered with a high dosage of BYF extract. For consistency with the efficacy experiment, the serum was collected at 10 min, 30 min, 1 h, 2 h, and 4 h after the last BYF administration (after 7 d of continuous oral administration of BYF extract). Based on the retention time and HRMS spectra, we identified 79 compounds in rat serum after oral BYF administration: 34 flavonoids, 14 lignans, 7 alkaloids, one saponin, three organic acids, four saccharides, three lipids, four terpenoids, two amino acids, one coumarin, and six other compounds. Among them, 26 were identified by comparison with the standard materials (Table S2). The total ion chromatograms of rat serum at 1 h after the administration of BYF extract are shown in Supplementary Materials Figure S38. The 13 main components of BYF in rat serum were quantified; endogenous compounds and compounds with insufficient contents were not measured. The quantitative results are shown in Figure 2(b) and Figure S39, and the standard curves and linear ranges are shown in Table S3. Among the BYF components detected in serum, schisandrin B, schisantherin A, and schisantherin B had the highest contents. Among the validated potential antioxidants (Table 2), hesperidin, and naringenin were detected in rat serum (Table 3 and Table S2); however, they are in trace amounts and the concentrations were not obtained. Rosmarinic acid was not found in serum after oral administrated of BYF, Thus, although rosmarinic acid showed the best antioxidant activity, it may not be the major active component in BYF due to its poor absorption or low content. Calycosin most likely to be responsible for the antioxidant effect of BYF *in vivo*, because it showed a high content in serum. The serum concentration–time curve of calycosin is shown in Figure 2(c). The serum concentration of calycosin reached its highest level at 10 min after the oral administration of BYF, and calycosin was almost cleared *in vivo* after 1 h. Based on the above results, the efficacy and mechanism of calycosin in the treatment of COPD *in vivo* are worthy of further study.

## 4. Conclusion

In this work, we first identified 189 compounds from the BYF extract. An offline DPPH-UHPLC Q-Extractive Orbitrap MS/MS strategy was developed to rapidly screen the antioxidants in BYF. Rosmarinic acid and calycosin showed high radical scavenging activities in both DPPH and ABTS assays. We detected a high content of calycosin in rat serum after the oral administration of BYF, suggesting that calycosin might be the key antioxidant compound in BYF for the treatment of COPD *in vivo*.

## Figures and Tables

**Table 1:** Chemical identification of BYF by UHPLC-Q-extractive Orbitrap MS/MS.

No.	RT	Formula	Identification	*m/z* [M + H]^+^	Error (ppm)	MS/MS	Origin
1	1.43	C_7_H_6_O_3_	4-Hydroxybenzoic acid isomer	139.0386	2.68	121.0281[M + H-H_2_O]^+^; 95.04889[M + H-CO_2_]^+^	PF
2	1.45	C_7_H_12_O_6_	Quinic acid^R^	193.0702	2.42	147.0650[M + H-CH_2_O-O]^+^; 129.0544[M + H-CH_2_O-O-H_2_O]^+^; 111.0439[M + H-CH_2_O-O-2H_2_O]^+^	EH
3	1.49	C_36_H_36_N_2_O_8_	Lyciumamide B	625.2541	0.55	607.2366[M + H-H_2_O]^+^; 589.2355^CFM_ID^	LF
4	1.51	C_7_H_6_O_5_	Gallic acid isomer	171.0283	2.94	153.0199[M + H-H_2_O]^+^; 127.0386[M + H-CO_2_]^+MB^	PRR/AJH
5	1.55	C_7_H_6_O_3_	4-Hydroxybenzoic acid	139.0386	2.68	121.0281[M + H-H_2_O]^+^; 95.0490[M + H-CO_2_]^+^	PF
6	1.75	C_13_H_18_O_7_	Isosalicin	287.1112	4.65	125.0594[M + H-glu]^+^	PRR
7	1.95	C_6_H_6_O_2_	5-Methyl furan aldehyde	111.0438	2.33	83.0489[M + H-CO]^+^	SCF
8	1.95	C_9_H_11_NO_2_	Phenylalanine isomer	166.0860	1.55	91.0539^MoNA^	PF
9	1.97	C_9_H_8_O_3_	p-Hydroxy-cinnamic acid	165.0543	1.95	121.0635[M + H-CO_2_]^+ MoNA^	LF, PF
10	2.22	C_5_H_7_NO_3_	Pyroglutamic acid^R^	130.0499	−0.24	84.0442	PF
11	11.23	C_14_H_16_O_9_	Bergenin	329.0880	−3.94	311.0752[M + H-H_2_O]^+^; 293.0647[M + H-2H_2_O]^+^; 275.0543[M + H-3H_2_O]^+^; 263.0543; 233.0439	AJH
12	11.69	C_16_H_18_O_9_	Cis-Cryptochlorogenic acid/trans-Cryptochlorogenic acid/Chlorogenic acid	355.1045	−6.05	163.0376^MB^	EH
13	11.82	C_39_H_50_O_26_	Quercetin-rha-tri-hex	935.2681	−1.92	303.0463[M + H-rha-3hex]^+^	LF
14	11.96	C_23_H_28_O_12_	Oxypaeoniflorin^R^	497.1668	−2.92	335.1158[M + H-glu]^+^; 133.0636; 121.0282	PR
15	12.00	C_17_H_26_O_10_	Loganin^R^	391.1616	−4.43	229.1065[M + H-glu]^+^; 197.0808; 179.0699	PF, AJH/AJH
16	16.15	C_27_H_30_O_14_	Apigenin-rha-glu	579.1729	−3.58	433.1132[M + H-rha]^+^; 271.0596[M + H-rha-glu]^+^	FTB, EH
17	16.21	C_32_H_38_O_16_	Hexandraside E	679.2266	−4.92	517.1617[M + H-glu]^+^; 355.1173[M + H-2glu]^+^; 299.0549[M + H-2glu-isobutenyl]^+^	EH
18	16.23	C_21_H_20_O_11_	Kaempferol-3-O-gal	449.1095	−3.71	287.0539[M + H-glu]^+^; 263.0550; 245.0442	AJH, EH/FTB, EH
19	16.35	C_23_H_28_O_11_	Albiflorin	481.1723	−3.88	319.1169[M + H-glu]^+^	PR
20	16.55	C_27_H_30_O_14_	Rhoifolin^R^	579.1729	−3.58	433.1132[M + H-rha]^+^; 271.0596[M + H-rha-glu]^+^	EH
21	16.72	C_22_H_22_O_10_	Calycosin-7-O-glu or its isomer (a)	447.1287	−0.28	285.0749[M + H-glu]^+^	AR
22	16.79	C_28_H_34_O_15_	Hesperidin^R^	611.1998	−4.51	465.1387[M + H-rha]^+^; 303.0860[M + H-rha-glu]^+^; 177.0545; 153.0181; 85.0283	CRP
23	16.81	C_18_H_16_O_7_	Dihydroxy-trimethoxyflavone	345.0985	−4.71	287.0555[M + H-2CH_3_-CO]^+^; 153.0179	CRP
24	16.81	C_22_H_24_O_11_	Hesperetin-7-O-glu or its isomer (a)	465.1396	−1.00	303.086[M + H-glu]^+^	GRR
25	16.89	C_21_H_22_O_10_	Prunin	435.1294	−1.90	417.0648[M + H-H_2_O]^+^; 343.1283; 273.0755[M + H-glu]^+^	CRP
26	16.93	C_18_H_16_O_8_	Rosmarinic acid^R^	361.0907	3.04	163.0384; 145.0273; 135.0429	PF/CRP
27	16.97	C_33_H_53_NO_8_	Sibelicin glycoside	592.3843	0.16	574.3731[M + H-H_2_O]^+^; 430.3316[M + H-glu]^+^; 412.3208[M + H-glu-H_2_O]^+^	FTB
28	17.09	C_22_H_22_O_11_	Diosmetin-6-C-glu/Pratensein-7-O-glu (a)	463.1247	−2.62	301.0703[M + H-glu]^+^	CRP/AR
29	17.15	C_21_H_20_O_10_	Apigenin-8-C-glu	433.1146	−3.88	271.0602[M + H-glu]^+^	CRP
30	17.19	C_28_H_32_O_15_	Diosmin^R^	609.1825	−1.81	463.1224[M + H-rha]^+^;301.0696[M + H-rha-glu]^+^;286.0461[M + H-rha-glu-CH_3_]^+^	CRP
31	17.31	C_27_H_41_NO_3_	Peimisine isomer (a)	428.3171	−2.76	410.3067[M + H-H_2_O]^+^	FTB
32	17.43	C_17_H_14_O_8_	Tetrahydroxy-dimethoxyflavone (a)	347.0753	2.44	287.0543[M + H-2OCH_3_]^+^	CRP
33	17.47	C_21_H_18_O_11_	Apigenin-7-O-gluA	447.0931	−2.04	271.0589[M + H-gluA]^+^; 167.0556	PF
34	17.72	C_27_H_41_NO_3_	Peimisine^R^	428.3171	−2.76	410.3026[M + H-H_2_O]^+^	FTB
35	17.86	C_32_H_38_O_15_	Des-O-methylicariin/epimedoside A	663.2299	−2.35	517.1711[M + H-rha]^+^; 355.1168[M + H-rha-glu]^+^; 299.0544[M + H-rha-glu-isobutenyl]^+^	EH
36	17.94	C_22_H_22_O_11_	Diosmetin-6-C-glu/pratensein-7-O-glu (b)	463.1242	1.54	301.0701[M + H-glu]^+^	CRP/AR
37	18.00	C_15_H_16_O_4_	Meranzin/isomeramazin (a)	261.1117	1.67	189.0547; 131.0487^MB^	CRP
38	18.30	C_16_H_14_O_6_	Hesperetin	303.0875	−3.92	177.0547; 171.0288; 153.0182 [24]	CRP
39	18.30	C_22_H_24_O_11_	Hesperetin-7-O-glu or its isomer (b)	465.1396	−1.00	303.0856[M + H-glu]^+^	GRR
40	18.52	C_27_H_41_NO_3_	Peimisine isomer (b)	428.3171	−2.76	410.3055[M + H-H_2_O]^+^	FTB
41	19.03	C_33_H_55_O_8_N	Zhebeininoside	594.4004	−0.60	576.3881[M + H-H_2_O]^+^; 414.3357[M + H-H_2_O-glu]^+^	FTB
42	19.17	C_27_H_45_NO_3_	Peimine A^R^	432.3473	−0.81	414.3361[M + H-H_2_O]^+^; 398.3082	FTB
43	19.91	C_27_H_41_NO_3_	Peimisine isomer (c)	428.3171	−2.76	410.2988[M + H-H_2_O]^+^; 337.2122	FTB
44	20.15	C_27_H_41_O_4_N	Peimisine nitrogen oxide	444.3102	1.43	398.3026[M + H-CH_2_O-O]^+^; 98.0961	FTB
45	20.23	C_27_H_41_NO_3_	Peimisine isomer (d)	428.3171	−2.76	410.2950[M + H-H_2_O]^+^	FTB
46	21.17	C_9_H_10_O_3_	Ethyl 4-hydroxybenzoate	167.0700	1.63	123.0436[M + H-C_2_H_2_O]^+^	EH
47	21.2	C_23_H_26_O_10_	Lactiflorin	463.1588	2.32	167.0701; 123.0441 [25]	PR
48	21.26	C_15_H_12_O_5_	Naringenin isomer (a)	273.0750	2.76	247.0427 [24]	CRP
49	21.32	C_27_H_43_NO_3_	Peiminine B^R^	430.3306	2.26	412.3206[M + H-H_2_O]^+^; 396.2866	FTB
50	21.53	C_39_H_50_O_20_	Epimedin A (hexandraside F) isomer	839.2953	1.81	677.2344[M + H-glu]^+^; 531.1817[M + H-glu-rha]^+^; 369.1326[M + H-2glu-rha]^+^; 313.0698[M + H-2glu-rha-isobutenyl]^+^	EH
51	21.78	C_33_H_53_NO_7_	Yibeinoside A or its isomer (a)	576.3880	2.57	414.3339[M + H-glu]^+^	FTB
52	21.80	C_9_H_10_O_3_	Paeonol isomer	167.0700	1.63	149.0582[M + H-H_2_O]^+^; 121.0644[M + H-H_2_O-CO]^+^	PRR
53	22.20	C_18_H_19_NO_4_	N-E-feruloyl tyramine	314.1377	3.14	177.0543; 145.0283; 121.0646	LF
54	22.26	C_27_H_30_O_11_	Neoicariin/wushanicariin/icariside I or their isomer (a)	531.1852	1.68	369.1323[M + H-glu]^+^; 313.0701[M + H-glu-isobutenyl]^+^	EH
55	22.38	C_39_H_50_O_20_	Epimedin A (hexandraside F)^R^	839.2944	2.89	677.2318[M + H-glu]^+^; 531.1852[M + H-glu-rha]^+^; 369.1326[M + H-2glu-rha]^+^; 313.0697[M + H-2glu-rha-isobutenyl]^+^	EH
56	22.42	C_28_H_34_O_14_	Poncirin/didymin	595.2002	3.25	433.1456[M + H-glu]^+^; 287.0909[M + H-glu-rha]^+^; 171.0285; 153.0180	CRP
57	22.45	C_22_H_22_O_9_	Ononin	415.1376	0.11	283.0845[M + H-glu]^+^; 219.0253; 183.0275; 132.0433; 89.0590	AR
58	22.51	C_16_H_12_O_5_	Calycosin^R^	285.0748	3.34	171.0275; 161.0595	AR
59	22.51	C_28_H_32_O_14_	Robinia pseudoxanthin-7-O-rutinoside	593.1850	2.50	447.1188[M + H-rha]^+^; 285.075[M + H-rha-glu]^+^	PF
60	22.98	C_38_H_48_O_19_	Epimedin B^R^	809.2841	2.67	677.2428[M + H-xyl]^+^; 531.1852[M + H-xyl-rha]^+^; 369.1324[M + H-xyl-rha-glu]^+^; 313.0698[M + H-xyl-rha-glu-isobutenyl]^+^	EH
61	22.98	C_27_H_30_O_11_	Neoicariin/wushanicariin/icariside I or their isomer (b)	531.1852	1.68	369.1326[M + H-glu]^+^; 313.0699[M + H-glu-isobutenyl]^+^	EH
62	23.13	C_33_H_53_NO_7_	Yibeinoside A or its isomer (b)	576.3878	2.92	414.3355[M + H-glu]^+^	FTB
63	23.26	C_27_H_30_O_11_	Neoicariin/wushanicariin/icariside I or their isomer (c)	531.1852	1.68	369.1328[M + H-glu]^+^; 313.0699[M + H-glu-isobutenyl]^+^	EH
64	23.28	C_39_H_50_O_19_	Epimedin C (baohuside VI)^R^	823.2999	2.44	677.2422[M + H-rha]^+^; 531.1848[M + H-2rha]^+^; 369.1324[M + H-2rha-glu]^+^; 313.0698[M + H-3rha-glu]^+^	EH
65	23.34	C_27_H_41_NO_3_	Peimisine isomer (e)	428.3171	−2.76	410.3058[M + H-H_2_O]^+^	FTB
66	23.40	C_30_H_34_O_17_	Sudachiin B/C	667.1860	1.32	361.0916; 346.0653; 315.0478 [24]	CRP
67	23.52	C_27_H_45_NO_3_	Isopeimine A	432.3473	0.05	414.3356[M + H-H_2_O]^+^	FTB
68	23.58	C_21_H_20_O_6_	Anhydroicaritin or its isomer (a)	369.1328	1.26	323.0738; 313.0687[M + H-isobutenyl]^+^	EH
69	23.60	C_33_H_40_O_15_	Icariin^R^	677.2423	2.51	531.1846[M + H-rha]^+^; 369.1322[M + H-rha-glu]^+^; 311.0697[M + H-rha-glu-isobutenyl]^+^	EH
70	23.60	C_27_H_30_O_11_	Neoicariin/wushanicariin/icariside I or their isomer (d)	531.1852	1.68	369.1323[M + H-glu]^+^; 313.0698[M + H-glu-isobutenyl]^+^	EH
71	23.68	C_47_H_78_O_19_	Astragaloside V/VI/VII	947.5190	2.12	437.3372; 419.3230 [26]	AR
72	23.69	C_36_H_62_O_9_	20(*R*)-Ginsenoside Rh_1_	639.4458	1.35	405.3482; 423.3584; 441.368 [27]	GRR
73	23.72	C_22_H_22_O_10_	Calycosin-7-O-glu or its isomer (b)	447.1287	−0.28	285.0751[M + H-glu]^+^; 149.0223; 89.0594	AR
74	23.77	C_33_H_55_NO_7_	Hupeheninoside	578.4039	2.13	416.3525[M + H-glu]^+^	FTB
75	23.85	C_15_H_12_O_5_	Naringenin isomer (b)	273.0750	2.76	247.0638; 171.0307; 153.0204 [2]	CRP
76	23.87	C_41_H_52_O_21_	Epimedin I	881.3035	−4.38	531.1829[M + H-glu-acetyl rha]^+^; 369.1328[M + H-glu-acetyl rha-glu]^+^; 313.0697[M + H-glu-acetyl rha-glu-isobutenyl]^+^	EH
77	23.91	C_27_H_32_O_11_	Icaritin-3-O-rha	533.2007	1.95	387.1393[M + H-rha]^+^; 369.1314[M + H-rha-H_2_O]^+^; 313.0701[M + H-rha-H_2_O-isobutenyl]^+^	EH
78	23.99	C_27_H_43_NO_3_	Peiminine B isomer	430.3306	2.26	412.3210[M + H-H_2_O]^+^	FTB
79	24.05	C_48_H_82_O_18_	Ginsenoside Re^R^	947.5531	4.53	325.1117	GRR
80	24.07	C_54_H_92_O_23_	Ginsenoside Rb1^R^	1109.6097	0.47	487.1687; 425.3734; 325.1105	GRR
81	24.09	C_40_H_50_O_20_	Sempervirenoside B	851.2945	2.73	369.1315[M + H-glu-rha (OAc)-xyl]^+^; 313.0700[M + H-glu-rha (OAc)-xyl-isobutenyl]^+^	EH
82	24.21	C_33_H_40_O_18_	Melitidin	725.2269	2.54	419.1328; 389.0858; 361.0910 [24]	CRP
83	24.23	C_26_H_28_O_11_	Epimedoside C	517.1690	2.79	355.1173[M + H-glu]^+^; 299.0547[M + H-glu-isobutenyl]^+^	EH
84	24.2	C_53_H_90_O_22_	Ginsenoside Rb2/ginsenoside Rc/ginsenoside Rb3	1079.6003	−0.6	457.1532; 425.3733; 407.3638; 325.1110 [27]	GRR
85	24.31	C_53_H_90_O_22_	Ginsenoside Rb2/ginsenoside Rc/ginsenoside Rb3	1079.5966	2.83	457.1523; 425.3726; 325.1110	GRR
86	24.35	C_10_H_14_O	Perillaldehyde^R^	151.1114	2.28	123.0438; 95.0489	PF
87	24.37	C_20_H_18_O_6_	Desmethylanhydroicaritin or its isomer (a)	355.1197	−5.89	299.0546[M + H-isobutenyl]^+^	EH
88	24.37	C_9_H_10_O_3_	Paeonol	167.0698	2.83	149.0579; 121.0641	GRR
89	24.39	C_36_H_56_O_9_	Calenduloside E	633.3981	2.55	439.3530 [27]	GRR
90	24.39	C_48_H_76_O_19_	Ginsenoside Ro	957.5087	−3.50	439.3530; 414.3343 [27]	GRR
91	24.41	C_27_H_43_NO_2_	Ebeiedinone/delavinone/zhebeirine (puqiedinone) (a)	414.3354	3.04	396.3243[M + H-H_2_O]^+^	FTB
92	24.43	C_41_H_68_O_14_	Astragaloside Iv^R^	785.4669	1.64	587.3879; 455.3473; 437.3373; 419.3258	AR
93	24.47	C_43_H_54_O_22_	Epimedokoreanoside I	923.3163	1.79	719.2438[M + H-glu (OAc)]^+^; 531.1782[M + H-glu (OAc)-rha (OAc)]^+^; 369.1328[M + H-glu (OAc)-rha (OAc)-glu]^+^; 313.0699[M + H-glu (OAc)-rha (OAc)-glu-isobutenyl]^+^	EH
94	24.5	C_30_H_32_O_12_	Benzoylpaeoniflorin/paeonivayin or their isomer (a)	585.1964	0.43	123.0432 [25]	PR
95	24.51	C_15_H_12_O_5_	Naringenin^R^	273.0750	2.76	247.0639; 229.0517; 171.0307; 153.0183; 147.0442	CRP
96	24.51	C_15_H_24_O	Spathulenol	221.1896	1.78	203.1772[M + H-H_2_O]^+^	EH
97	24.59	C_15_H_10_O_5_	Apigenin^R^	271.0593	2.96	245.0643[M + H-O]^+^; 229.0854; 177.0542; 121.0281; 107.0488	PF
98	24.61	C_35_H_42_O_16_	Epimedokoreanoside II/sagittatoside C	719.2521	3.43	369.1332[M + H-glu-rha (OAc)]^+^; 313.0700[M + H-glu-rha (OAc)-isobutenyl]^+^	EH
99	24.63	C_31_H_36_O_14_	Ikarisoside F	633.2161	2.66	355.1166[M + H-rha-xyl]^+^; 299.0546[M + H-rha-xyl-isobutenyl]^+^	EH
100	24.69	C_27_H_43_NO_2_	Ebeiedinone/delavinone/zhebeirine (puqiedinone) (b)	414.3354	3.04	396.3246[M + H-H_2_O]^+^	FTB
101	24.69	C_33_H_53_NO_7_	Yibeinoside A or its isomer (c)	576.3872	3.96	558.3722[M + H-H_2_O]^+^; 396.3239[M + H-H_2_O-glu]^+^	FTB
102	24.73	C_15_H_24_O	Caryophyllene oxide or its isomer (a)	221.1896	1.78	203.1786[M + H-H_2_O]^+CFM-ID^	PF/AJH
103	24.81	C_36_H_62_O_9_	20(S)-ginsenoside Rh_1_	639.4478	2.66	441.3716; 423.3512; 405.3509	GRR
104	24.81	C_16_H_12_O_6_	Chrysoeriol	301.0698	2.88	286.0468[M + H-CH_3_]^+^; 258.0519[M + H-CH_3_-CO_2_]^+^; 177.0544; 153.0182 [28]	PF, AR
105	24.83	C_15_ H_22_ O	Chamigrenal isomer or its isomer (a)	219.1737	2.94	203.1425; 121.1009^CFM-ID^	SCF
106	24.93	C_45_H_56_O_23_	Caohuoside A (epimedin L)/caohuoside B/epimedin K (korepimedoside B)	965.3265	2.09	369.1313[M + H-glu (2OAc)-rha (OAc)-glu]^+^; 313.0694[M + H-glu (2OAc)-rha (OAc)-glu-isobutenyl]^+^	EH
107	24.93	C_18_H_16_O_8_	Trihydroxy-trimethoxyflavone	361.0907	3.04	331.0441[M + H-2CH_3_]^+^	CRP
108	24.97	C_43_H_70_O_15_	Astragaloside II/isoastragaloside II	827.4761	3.20	629.3947; 175.0599[Xyl (OAc)+H]^+^ 157.0494[Xyl (OAc)+H-H_2_O]^+^ [26]	AR
109	24.99	C_18_H_16_O_9_	Tetrahydroxy-trimethoxyflavone	377.0857	2.68	362.0611[M + H-CH_3_]^+^; 347.0385[M + H-2CH_3_]^+^; 319.0427[M + H-2CH_3_-CO]^+^	CRP
110	25.01	C_26_H_28_O_10_	Baohuoside II	501.1738	3.45	355.1169[M + H-rha]^+^; 299.0544[M + H-rha-isobutenyl]^+^; 121.0274	EH
111	25.01	C_20_H_18_O_6_	Desmethylanhydroicaritin or its isomer (b)	355.1197	−5.89	299.0546[M + H-isobutenyl]^+^	EH
112	25.13	C_17_H_14_O_8_	Tetrahydroxy-dimethoxyflavone (b)	347.0753	2.44	287.0533[M + H-2OCH_3_]^+^	CRP
113	25.15	C_21_H_20_O_6_	Anhydroicaritin or its isomer (b)	369.1328	1.26	313.0700[M + H-isobutenyl]^+^	EH
114	25.15	C_33_H_40_O_15_	Anhydroicaritin-3-O-Î“-L-rhamnosyl-7-O-Î”-D-Glucopyranoside/sagittatoside A	677.2426	2.07	369.1324[M + H-glu-rha]^+^; 313.0700[M + H-glu-rha-isobutenyl]^+^; 225.1012	EH
115	25.21	C_30_H_32_O_12_	Benzoylpaeoniflorin/paeonivayin or their isomer (b)	585.1951	2.66	123.0437 [25]	PR
116	25.23	C_48_H_78_O_18_	Soyasaponin I	943.5243	1.90	441.3683; 423.3577^CFM-ID^	AR
117	25.34	C_16_H_12_O_4_	Formononetin^R^	269.0806	0.88	254.0566; 237.0544; 213.0908;	AR
118	25.34	C_19_H_20_O_7_	Monohydroxy-tetramethoxyflavanone	361.1273	2.44	211.0574 [24]	CRP
119	25.34	C_20_H_22_O_7_	Pentamethoxyflavanone (a)	375.1434	1.15	211.0588 [24]	CRP
120	25.38	C_21_H_20_O_6_	Anhydroicaritin or its isomer (c)	369.1328	1.26	313.7[M + H-rha-isobutenyl]^+^	EH
121	25.38	C_32_H_38_O_14_	Sagittatoside B	647.2322	1.91	515.1830[M + H-xyl]^+^; 369.1330[M + H-xyl-rha]^+^; 313.0702[M + H-xyl-rha-isobutenyl]^+^	EH
122	25.42	C_33_H_40_O_14_	2″-O-rhamnosylicariside II/anhydroicaritin 3-O-2″-rha-rha	661.2476	2.25	515.1898[M + H-rha]^+^; 369.1328[M + H-2rha]^+^; 313.0699[M + H-2rha-isobutenyl]^+^	EH
123	25.54	C_27_H_41_NO_3_	Peimisine isomer (f)	428.3171	−2.76	410.3037[M + H-H_2_O]^+^	FTB
124	25.56	C_15_H_16_O_4_	Meranzin/isomeramazin (b)	261.1117	1.67	189.0536; 131.0483^MB^	CRP
125	25.59	C_42_H_72_O_13_	Ginsenoside Rg2^R^	785.5023	2.89	441.3702; 423.3601 [27]	GRR
126	25.66	C_20_H_20_O_7_	Isosinensetin^R^	373.1275	1.83	357.0966; 343.0877; 315.0858	CRP
127	25.72	C_27_H_30_O_11_	Neoicariin/wushanicariin/icariside I or their isomer (e)	531.1852	1.68	369.1325[M + H-glu]^+^; 313.0700[M + H-isobutenyl]^+^	EH
128	25.78	C_45_H_72_O_16_	Astragaloside I/isoastragaloside I	869.4894	−0.10	157.0482[Xyl (OAc) + H-H_2_O]^+^ [26]	AR
129	25.86	C_21_H_22_O_8_	Hexamethoxyflavone (a)	403.1381	1.6	388.1140; 373.0914; 358.0642; 327.0588 [24]	CRP
130	25.92	C_26_H_32_O_8_	Deacetylnomilin	473.2163	1.47	161.0598 [24]	CRP
131	25.94	C_36_H_60_O_8_	Ginsenoside Rk3/Rh4	621.4347	2.25	405.3362 [28]	GRR
132	26.02	C_21_H_20_O_6_	Anhydroicaritin or its isomer (d)	369.1328	1.26	313.0699[M + H-isobutenyl]^+^	EH
133	26.02	C_27_H_30_O_10_	Icariside II (baohuside I)	515.1907	0.92	369.1328[M + H-rha]^+^; 313.0701[M + H-rha-isobutenyl]^+^	EH
134	26.16	C_20_H_20_O_7_	Sinensetin	373.1279	0.75	357.0968; 343.0805; 329.1013; 297.0734 [24]	CRP
135	26.24	C_11_H_16_O	Jasmonane	165.1273	0.56	123.1172^MoNA^	PF
136	26.26	C_22_H_26_O_6_	Gomisin L1 or its isomer (a)	387.1800	0.56	287.0549[M + H-C_5_H_10_-2CH_3_]^+^	SCF
137	26.26	C_11_H_14_O_2_	Methyl eugenol^R^	179.1067	−0.25	151.0752; 116.9718	PF
138	26.28	C_19_H_18_O_6_	Tetramethyl-O-scutellarein/tetramethyl-O-isoscutellarein/tetramethoxyflavone (a)	343.1173	0.92	313.0701[M + H-2CH_3_]^+^; 285.0751[M + H-2CH_3_-CO]^+^	CRP
139	26.34	C_20_H_22_O_7_	Pentamethoxyflavanone (b)	375.1436	0.61	211.0596; 150.0304 [24]	CRP
140	26.42	C_18_H_39_NO_3_	D-ribo-phytosphingosine^R^	318.2998	1.48	300.2895[M + H-H_2_O]^+^; 282.2784[M + H-2H_2_O]^+^;	PF
141	26.48	C_24_H_32_O_7_	Schisandrol A^R^	433.2128	−0.05	415.2106[M + H-H_2_O]^+^; 400.1871[M + H-H_2_O-CH_3_]^+^; 384.1922; 369.1688; 338.1504	SCF
142	26.57	C_19_H_18_O_8_	Rosmarinic acid methylester	375.1076	-0.42	135.0435	PF
143	26.61	C_21_H_22_O_8_	Nobiletin^R^	403.1384	0.86	388.1145[M + H-CH_3_]^+^; 373.0912[M + H-2CH_3_]^+^; 358.0672[M + H-3CH_3_]^+^; 327.0857; 301.0701	CRP
144	26.63	C_18_H_32_O_3_	13-Hydroxy-9,11-octadecadienoic acid or its isomer (a)	297.2416	2.77	279.2318[M + H-H_2_O]^+^; 261.2202[M + H-2H_2_O]^+^	AR
145	26.63	C_18_H_30_O_2_	a-Linolenic acid or its isomer (a)	279.2308	3.80	261.2190[M + H-H_2_O]^+^; 149.0232; 121.0282^MoNA^	PF/EH
146	26.83	C_19_H_18_O_6_	Tetramethyl-O-scutellarein/tetramethyl-O-isoscutellarein/tetramethoxyflavone (b)	343.1174	0.63	313.0701[M + H-2CH_3_]^+^; 285.0750[M + H-2CH_3_-CO]^+^	CRP
147	26.97	C_22_H_24_O_9_	3,5,6,7,8,3′,4′-heptamethoxyflavone	433.1488	1.18	418.1244[M + H-CH_3_]^+^; 403.1016[M + H-2CH_3_]^+^; 388.0700[M + H-3CH_3_]^+^; 373.0541[M + H-4CH_3_]^+^ [24]	CRP
148	27.07	C_18_H_32_O_3_	13-hydroxy-9,11-octadecadienoic acid or its isomer (b)	297.2416	2.77	279.2313[M + H-H_2_O]^+^; 261.2217[M + H-2H_2_O]^+^	AR
149	27.17	C_42_H_70_O_12_	Ginsenoside Rg5/Rk1	767.4932	1.05	605.4375[M + H-glu]^+^; 439.3837; 425.3744; 407.3635	GRR
150	27.29	C_23_H_28_O_7_	Schisandrol B/epigomisin O	417.1900	1.87	399.1796[M + H-H_2_O]^+^; 368.1609; 299.0598; 119.0854	SCF
151	27.29	C_20_H_20_O_7_	Tangeretin^R^	373.1277	1.29	343.0807[M + H-2CH_3_]^+^; 297.0753; 229.0328; 135.0437	CRP
152	27.39	C_28_H_34_O_9_	Schisantherin C (angeloylgomisin P) or its isomer (a)	515.2258	3.42	385.1667 [M + H-C_4_H_6_COOH-CH_2_O]^+^; 355.1537[M + H-C_4_H_6_COOH-2CH_2_O]^+^; 339.1198; 316.0930; 301.0690	SCF
153	27.75	C_21_H_22_O_8_	Hexamethoxyflavone (b)	403.1380	1.85	388.1145[M + H-CH_3_]^+^; 373.0913[M + H-2CH_3_]^+^; 355.0803[M + H-CH_3_-H_2_O]^+^; 327.0856[M + H-CH_3_-CO]^+^ [24]	CRP
154	27.89	C_28_H_36_O_8_	Angeloylgomisin H or its isomer (a)	501.2473	1.99	483.2372[M + H-H_2_O]^+^; 437.1929; 401.1953[M + H-C_4_H_6_COOH]^+^	SCF
155	27.93	C_23_H_28_O_6_	Schisandrin B (*γ*-schisandrin) isomer (a)	401.1945	3.41	386.1718[M + H-CH_3_]^+^; 370.1762; 355.1531; 345.1320	SCF
156	27.95	C_20_H_22_O_7_	Pentamethoxyflavanone (c)	375.1432	1.68	357.0634; 211.0595 [24]	CRP
157	28.01	C_28_H_34_O_8_	Benzoyl isogomisin O or its isomer	499.2328	−0.31	483.2370[M + H-O]^+^; 451.2118[M + H-O-CH_3_OH]^+^	SCF
158	28.09	C_15_ H_22_ O	Chamigrenal isomer or its isomer (b)	219.1737	2.94	203.1429^CFM-ID^	SCF
159	28.12	C_23_H_28_O_6_	Schisandrin B (*γ*-schisandrin) isomer (b)	401.1945	3.41	386.1718[M + H-CH_3_]^+^; 370.1764; 355.1530; 345.1324	SCF
160	28.14	C_28_H_36_O_8_	Angeloylgomisin H or its isomer (b)	501.2469	2.79	483.2372[M + H-H_2_O]^+^; 437.1929; 401.1955[M + H-C_4_H_6_COOH]^+^; 370.1768	SCF
161	28.26	C_22_H_26_O_6_	Gomisin L1 or its isomer (b)	387.1800	0.56	287.0548[M + H-C_5_H_10_-2CH_3_]^+^	SCF
162	28.36	C_18_H_30_O_2_	a-Linolenic acid or its isomer (b)	279.2308	3.80	263.2362; 149.0232; 121.0282; 95.0854^MoNA^	PF/EH
163	28.46	C_21_H_22_O_9_	Natsudaidai	419.1326	2.53	389.0862[M + H-2CH_3_]^+^; 361.0892[M + H_2_CH_3_CO]^+^; 299.0611; 181.0855	CRP
164	28.46	C_22_H_24_O_6_	Schisandrin C isomer	385.1631	3.81	355.1545[M + H-CH_2_O]^+^; 337.1415[M + H-CH_2_O-H_2_O]^+^; 316.0928	SCF
165	28.52	C_30_H_34_O_8_	Benzoyl gomisin H	523.2314	2.38	505.2202[M + H-H_2_O]^+^; 401.1912; 370.1731	SCF
166	28.52	C_25_H_26_O_6_	Epimedokoreanin B	423.1790	2.88	311.0522[M + H-2isobutenyl]^+^	EH
167	28.52	C_23_H_30_O_6_	Gomisin K1	403.2105	2.52	388.1869[M + H-CH_3_]^+^; 371.1848; 340.1656; 333.1236; 302.1237	SCF
168	28.56	C_30_H_48_O_4_	Corosolic acid^R^	473.3614	2.41	409.3441; 205.1585; 189.1642; 177.1634; 95.0853	PF
169	28.95	C_28_H_34_O_9_	Schisantherin B^R^	515.2266	1.87	415.1732[M + H-C_4_H_6_COOH]^+^	SCF
170	29.00	C_22_H_26_O_6_	Gomisin L1 or its isomer (c)	387.1800	0.56	355.1521[M + H-CH_3_OH]^+^; 317.1023[M + H-C_5_H_10_]^+;^ 287.0540[M + H-C_5_H_10_-2CH_3_]^+^	SCF
171	29.00	C_23_H_26_O_7_	Neoisostegane	415.1740	2.73	397.1630[M + H-H_2_O]^+^; 371.1483[M + H-CO_2_]^+^; 356.1243; 340.1299	SCF
172	29.00	C_23_H_30_O_6_	Schisanhenol	403.2105	2.52	388.1877[M + H-CH_3_]^+^; 371.1856[M + H-CH_3_-OH]^+^; 356.1614; 340.1665; 325.1429; 305.1322	SCF
173	29.04	C_30_H_32_O_9_	Schisantherin A^R^	537.2109	1.88	415.1724; 268.9779; 91.0565	SCF
174	29.06	C_15_ H_22_ O	Chamigrenal isomer or its isomer (c)	219.1737	2.94	203.1438^CFM-ID^	SCF
175	29.14	C_28_H_34_O_9_	Schisantherin C (angeloylgomisin P) or its isomer (b)	515.2258	3.42	385.1630 [M + H-C_4_H_6_COOH-CH_2_O]^+^; 355.1498[M + H-C_4_-H_6_COOH-2CH_2_O]^+^; 339.1175; 316.0931; 301.0695	SCF
176	29.30	C_22_H_26_O_6_	Gomisin L1 or its isomer (d)	387.1800	0.56	355.0541[M + H-CH_3_OH]^+^; 317.1023[M + H-C_5_H_10_]^+^; 287.0536[M + H-C_5_H_10_-2CH_3_]^+^	SCF
177	29.50	C_22_H_22_O_7_	Baohuosu	399.1425	3.34	355.1163[M + H-C_3_H_6_]^+^; 325.1052	EH
178	29.59	C_18_H_32_O_3_	13-Hydroxy-9,11-octadecadienoic acid or its isomer (c)	297.2416	2.77	279.2316[M + H-H_2_O]^+^; 261.2198[M + H-H_2_O]^+^	AR
179	29.63	C_28_H_34_O_9_	Schisantherin C (angeloylgomisin P) or its isomer (c)	515.2258	3.42	385.1630[M + H-C_4_H_6_COOH-CH_2_O]^+^; 355.1525[M + H-C_4_H_6_COOH-2CH_2_O]^+^; 339.1222; 316.0939; 301.0708	SCF
180	29.71	C_22_H_26_O_6_	Gomisin L1 or its isomer (e)	387.1800	0.56	355.1543[M + H-CH_3_OH]^+^; 317.1017[M + H-C_5_H_10_]^+^; 287.0540[M + H-C_5_H_10_-2CH_3_]^+^	SCF
181	29.71	C_19_H_20_O_6_	Tetramethoxyflavanone	345.1321	3.38	330.1086[M + H-CH_3_]^+^; 315.0858[M + H-2CH_3_]^+^; 297.0932[M + H-2CH_3_-H_2_O]^+^; 287.0883[M + H-2CH_3_-CO]^+^; 247.0438	CRP
182	29.96	C_15_ H_22_ O	Chamigrenal isomer or its isomer (d)	219.1737	2.94	203.1438; 149.0594; 135.0803; 121.1005^CFM-ID^	SCF
183	30.27	C_24_H_32_O_6_	Schisandrin A^R^	417.2258	3.28	402.2029[M + H-CH_3_]^+^; 386.2079[M + H-CH_3_-O]^+^; 371.1832[M + H-2CH_3_-O]^+^; 347.1481; 316.1296; 301.1062	SCF
184	30.92	C_18_H_30_O_2_	a-linolenic acid or its isomer (c)	279.2308	3.80	263.2366; 149.0231; 121.0282; 95.0853^MoNA^	PF/EH
185	31.1	C_23_H_28_O_6_	Schisandrin B^R^	401.1945	3.41	386.1717; 370.1769; 355.1532; 331.1166; 300.0985; 285.0753; 270.0878	SCF
186	31.16	C_15_H_24_	Trans-*α*-acacia	205.1943	3.81	107.0849; 93.0694; 69.0696^MoNA^	PF
187	31.18	C_15_H_24_O	Caryophyllene oxide or its isomer (b)	221.1894	2.69	203.1775[M + H-H_2_O]^+CFM-ID^	PF/AJH
188	31.22	C_12_H_16_O_7_	Arbutin^R^	273.0958	3.97	157.0121; 139.0016; 129.0180	SCF
189	31.75	C_22_H_24_O_6_	Schisandrin C^R^	385.1631	3.81	355.1518; 315.0834; 285.0753; 257.0812; 228.0695	SCF

R: standard references; MB: massban. MoNA: massbank of North America; CFM-ID: CFM-ID.

**Table 2:** ABTS radical scavenging activity, DPPH radical scavenging activity, and ferric-reducing antioxidant power (FRAP) of BYF.

FRAP (mmol FeSO_4_/g)		
BYF	0.51 ± 0.04	
	DPPH IC_50_ (*μ*g/mL)	ABTS IC_50_ (*μ*g/mL)
BYF	1136.36 ± 148.03	602.3533 ± 81.26
Rosmarinic acid	25.72 ± 1.02	19.00 ± 0.75
Calycosin	147.23 ± 25.12	19.34 ± 5.05
Hesperidin	940.32 ± 65.02	75.7 ± 0.62
Naringenin	—	177.44 ± 16.94
L-ascorbic acid	24.58 ± 0.32	26.10 ± 1.16

**Table 3:** Candidate antioxidants identified from BYF and rat serum after the oral administration of BYF (10 mM DPPH).

	Compound	Intensity reduced	Rat serum
21	Calycosin-7-O-glu or its isomer (a)	−100 ± 0%	−
36	Diosmetin-6-C-glu/pratensein-7-O-glu (b)	−100 ± 0%	−
53	N-E-feruloyl tyramine	−100 ± 0%	+
104	Chrysoeriol	−100 ± 0%	+
28	Diosmetin-6-C-glu/pratensein-7-O-glu (a)	−100 ± 0%	−
30	Diosmin	−100 ± 0%	−
26	Rosmarinic acid	−100 ± 0%	−
66	Sudachiin B/C	−100 ± 0%	−
32	Tetrahydroxy-dimethoxyflavone (a)	−100 ± 0%	−
112	Tetrahydroxy-dimethoxyflavone (b)	−100 ± 0%	−
109	Tetrahydroxy-trimethoxyflavone	−100 ± 0%	−
181	Tetramethoxyflavanone	−100 ± 0%	−
107	Trihydroxy-trimethoxyflavone	−100 ± 0%	−
172	Schisanhenol	−99.88 ± 0.11%	+
58	Calycosin	−99.19 ± 0.17%	+
39	Hesperetin-7-O-glu or its isomer (b)	−99.14 ± 0.86%	−
170	Gomisin L1 or its isomer (c)	−99.09 ± 0.79%	−
24	Hesperetin-7-O-glu or its isomer (a)	−98.87 ± 1.11%	+
22	Hesperidin	−97.92 ± 1.87%	+
127	Neoicariin/wushanicariin/icariside I or their isomer (e)	−97.78 ± 0.2%	−
38	Hesperetin	−97.23 ± 2.55%	+
180	Gomisin L1 or its isomer (e)	−95.7 ± 3.77%	−
83	Epimedoside C	−93.81 ± 0.8%	+
176	Gomisin L1 or its isomer (d)	−91.19 ± 7.72%	−
142	Rosmarinic acid methylester	−87.71 ± 1.01%	−
163	Natsudaidai	−77.87 ± 20.02%	−
161	Gomisin L1 or its isomer (b)	−72.78 ± 0.21%	−
167	Gomisin K1	−66.94 ± 4.97%	+
119	Pentamethoxyflavanone (a)	−60.01 ± 35.31%	−
136	Gomisin L1 or its isomer (a)	−56.9 ± 3.72%	+
95	Naringenin	−52.81 ± 2.96%	+
179	Schisantherin C (angeloylgomisin P) or its isomer (c)	−52.03 ± 5.84%	−
114	Anhydroicaritin-3-O-rhamnosyl-7-O-glucopyranoside/sagittatoside A	−48.26 ± 1.63%	+
98	Epimedokoreanoside II/sagittatoside C	−47.47 ± 14.34%	−
120	Anhydroicaritin or its isomer (c)	−46.99 ± 3.58%	−
99	Ikarisoside F	−46.02 ± 5.68%	−
121	Sagittatoside B	−45.64 ± 2.03%	+
149	Ginsenoside Rg5/Rk1	−44.74 ± 0.63%	−
110	Baohuoside II	−44.32 ± 4.95%	−
122	2″-O-rhamnosylicariside II/anhydroicaritin 3-O-2″-rha-rha	−43.86 ± 3.13%	+
133	Icariside II (baohuside I)	−41.97 ± 1.9%	+
132	Anhydroicaritin or its isomer (d)	−40.5 ± 0.89%	−
97	Apigenin	−39.92 ± 14.63%	+
113	Anhydroicaritin or its isomer (b)	−39.57 ± 3.19%	−
92	Astragaloside Iv	−39.11 ± 5.35%	−
93	Epimedokoreanoside I	−35.72 ± 8.33%	−
177	Baohuosu	−35.12 ± 2.68%	+
77	Icaritin-3-O-rha	−32.89 ± 6.35%	−
117	Formononetin	−32.3 ± 1.23%	+
124	Meranzin/isomeramazin (b)	−31.73 ± 6.19%	−
80	Ginsenoside Rb1	−31.42 ± 7.33%	−
178	13-hydroxy-9,11-octadecadienoic acid or its isomer (c)	−31.09 ± 10.58%	−
75	Naringenin isomer (b)	−30.76 ± 0.8%	−
129	Hexamethoxyflavone (a)	−28.6 ± 3.48%	+
150	Schisandrol B/epigomisin O	−26.94 ± 2.99%	+
33	Apigenin-7-O-gluA	−26.88 ± 8.86%	+
134	Sinensetin	−26.76 ± 1.67%	+
94	Benzoylpaeoniflorin/paeonivayin or their isomer (a)	−26.62 ± 4.87%	−
91	Ebeiedinone/delavinone/zhebeirine (puqiedinone) (a)	−26.21 ± 1.23%	+
65	Peimisine isomer (e)	−25.88 ± 5.23%	−
139	Pentamethoxyflavanone (b)	−25.33 ± 4.29%	−
147	3,5,6,7,8,3′,4′-Heptamethoxyflavone	−25.31 ± 3.13%	+
81	Sempervirenoside B	−24.59 ± 11.39%	−
151	Tangeretin	−24.37 ± 2.2%	+
126	Isosinensetin	−24.31 ± 1.4%	+
143	Nobiletin	−23.76 ± 0.63%	+
160	Angeloylgomisin H or its isomer (b)	−23.74 ± 3.76%	−
154	Angeloylgomisin H or its isomer (a)	−23.58 ± 3.47%	+
108	Astragaloside II/isoastragaloside II	−22.98 ± 5.24%	−
116	Soyasaponin I	−22.79 ± 7.26%	+
189	Schisandrin C	−22.79 ± 11.89%	+
100	Ebeiedinone/delavinone/zhebeirine (puqiedinone) (b)	−22.71 ± 4.35%	−
82	Melitidin	−22.65 ± 6.9%	+
44	Peimisine nitrogen oxide	−22.07 ± 3.99%	−
155	Schisandrin B (*γ*-schisandrin) isomer (a)	−21.68 ± 1.42%	+
153	Hexamethoxyflavone (b)	−21.31 ± 3.14%	−
43	Peimisine isomer (c)	−21.14 ± 10.74%	−
165	Benzoyl gomisin H	−20.24 ± 0.72%	−
71	Astragaloside V/VI/VII	−20.14 ± 21.7%	−

## Data Availability

The other data used in the manuscript are listed in supplementary materials.

## Supplementary Materials

Supplementary Materials The supporting data offered in supplementary materials are as follows: Table S1. Parallel reaction monitoring transitions for metabolites of Bufei Yishen formula included in the assay. Table S2. Chemical composition information of Bufei Yishen Formula Based on UHPLC-Q-Extractive Orbitrap MS. Table S3. Calibration curves, Linear ranges and LLOQs of the Bufei Yishen Formula compounds in serum. Figure S1. The MS/MS spectra of the reference standard of quinic acid. Figure S2. The MS/MS spectra of the reference standard of Pyroglutamic acid. Figure S3. The MS/MS spectra of the reference standard of oxypaeoniflorin. Figure S4. The MS/MS spectra of the reference standard of loganin. Figure S5. The MS/MS spectra of the reference standard of rhoifolin. Figure S6. The MS/MS spectra of the reference standard of Hesperidin. Figure S7. The MS/MS spectra of the reference standard of rosmarinic acid. Figure S8. The MS/MS spectra of the reference standard of diosmin. Figure S9. The MS/MS spectra of the reference standard of Peimisine. Figure S10. The MS/MS spectra of the reference standard of peimine A. Figure S11. The MS/MS spectra of the reference standard of Peiminine B. Figure S12. The MS/MS spectra of the reference standard of Epimedin A (Hexandraside F). Figure S13. The MS/MS spectra of the reference standard of Calycosin. Figure S14. The MS/MS spectra of the reference standard of Epimedin B. Figure S15. The MS/MS spectra of the reference standard of Epimedin C (Baohuside VI). Figure S16. The MS/MS spectra of the reference standard of Icariin. Figure S17. The MS/MS spectra of the reference standard of Ginsenoside Re. Figure S18. The MS/MS spectra of the reference standard of Ginsenoside Rb1. Figure S19. The MS/MS spectra of the reference standard of Perillaldehyde. Figure S20. The MS/MS spectra of the reference standard of astragaloside Iv. Figure S21. The MS/MS spectra of the reference standard of Naringenin. Figure S22. The MS/MS spectra of the reference standard of Apigenin. Figure S23. The MS/MS spectra of the reference standard of Formononetin. Figure S24. The MS/MS spectra of the reference standard of Ginsenoside Rg2. Figure S25. The MS/MS spectra of the reference standard of isosinensetin. Figure S26. The MS/MS spectra of the reference standard of Methyl eugenol. Figure S27. The MS/MS spectra of the reference standard of D-ribo-phytosphingosine. Figure S28. The MS/MS spectra of the reference standard of schisandrol A. Figure S29. The MS/MS spectra of the reference standard of Nobiletin. Figure S30. The MS/MS spectra of the reference standard of tangeretin. Figure S31. The MS/MS spectra of the reference standard of corosolic acid. Figure S32. The MS/MS spectra of the reference standard of Schisantherin B. Figure S33. The MS/MS spectra of the reference standard of Schisantherin A. Figure S34. The MS/MS spectra of the reference standard of Schisandrin A. Figure S35. The MS/MS spectra of the reference standard of Schisandrin B. Figure S36. The MS/MS spectra of the reference standard of Arbutin. Figure S37. The MS/MS spectra of the reference standard of Schisandrin C. Figure S38. A total ion chromatogram of the mix rat serum after administrated of BYF extract at 1 h. Figure S39. The content of Bufei Yishen Formula components in the rat serum.
