# Supplementary material for: Chemical Identification and Antioxidant Screening of Bufei Yishen Formula using an Offline DPPH Ultrahigh-Performance Liquid Chromatography Q-Extractive Orbitrap MS/MS
Source: Int J Anal Chem. 2022 Oct 15;2022:1423801. doi: 10.1155/2022/1423801 (PMC9588378; doi:10.1155/2022/1423801)
Supplement: Supplementary Materials — The supporting data offered in supplementary materials are as follows: Table S1. Parallel reaction monitoring transitions for metabolites of Bufei Yishen formula included in the assay. Table S2. Chemical composition information of Bufei Yishen Formula Based on UHPLC-Q-Extractive Orbitrap MS. Table S3. Calibration curves, Linear ranges and LLOQs of the Bufei Yishen Formula compounds in serum. Figure S1. The MS/MS spectra of the reference standard of quinic acid. Figure S2. The MS/MS spectra of the reference standard of Pyroglutamic acid. Figure S3. The MS/MS spectra of the reference standard of oxypaeoniflorin. Figure S4. The MS/MS spectra of the reference standard of loganin. Figure S5. The MS/MS spectra of the reference standard of rhoifolin. Figure S6. The MS/MS spectra of the reference standard of Hesperidin. Figure S7. The MS/MS spectra of the reference standard of rosmarinic acid. Figure S8. The MS/MS spectra of the reference standard of diosmin. Figure S9. The MS/MS spectra of the reference standard of Peimisine. Figure S10. The MS/MS spectra of the reference standard of peimine A. Figure S11. The MS/MS spectra of the reference standard of Peiminine B. Figure S12. The MS/MS spectra of the reference standard of Epimedin A (Hexandraside F). Figure S13. The MS/MS spectra of the reference standard of Calycosin. Figure S14. The MS/MS spectra of the reference standard of Epimedin B. Figure S15. The MS/MS spectra of the reference standard of Epimedin C (Baohuside VI). Figure S16. The MS/MS spectra of the reference standard of Icariin. Figure S17. The MS/MS spectra of the reference standard of Ginsenoside Re. Figure S18. The MS/MS spectra of the reference standard of Ginsenoside Rb1. Figure S19. The MS/MS spectra of the reference standard of Perillaldehyde. Figure S20. The MS/MS spectra of the reference standard of astragaloside Iv. Figure S21. The MS/MS spectra of the reference standard of Naringenin. Figure S22. The MS/MS spectra of the reference standard of Apigeni [file 1423801.f1.docx]

Table S1 Parallel reaction monitoring transitions for metabolites of Bufei Yishen formula included in the assay

| Compounds | Retention time | Precursor ion | Product ion | NCE |
| --- | --- | --- | --- | --- |
| Apigenin | 11.57 | 271.0593 | 153.0181 | 70 |
| Calycosin | 11.09 | 285.0748 | 270.0518 | 50 |
| Corosolic acid | 17.13 | 473.3614 | 205.1584 | 20 |
| Epimedin B | 11.08 | 809.2841 | 369.1323 | 30 |
| Isosinensetin | 11.86 | 373.1275 | 343.0805 | 40 |
| Peimine A | 5.43 | 432.3473 | 414.3359 | 50 |
| Peiminine B | 5.88 | 430.3306 | 412.3204 | 50 |
| Peimisine | 5.19 | 428.3171 | 114.0912 | 40 |
| Schisandrin A | 19.19 | 417.2258 | 316.1300 | 30 |
| Schisandrin B | 20.68 | 401.1945 | 300.0988 | 40 |
| Schisantherin A | 16.12 | 554.2379 | 415.1746 | 10 |
| Schisantherin B | 16.28 | 532.2534 | 415.1746 | 20 |
| Nobiletin | 12.87 | 403.1384 | 373.0912 | 40 |

NCE: Normalized collision energy

Table S2 Chemical composition information of Bufei Yishen Formula Based on UHPLC-Q-Extractive Orbitrap MS

| **Compound** | 10 min | 30 min | 1 h | 2 h | 4 h | RT | m/z (Expected) |
| --- | --- | --- | --- | --- | --- | --- | --- |
| 13-hydroxy-9,11-octadecadienoic acid or its isomer | +/+/+ | +/+/+ | +/+/+ | +/+/+ | +/+/+ | 26.63/27.07/29.59 | 297.2416 |
| 2"-O-Rhamnosylicariside II/Anhydroicaritin 3-O-2"-rha-rha | + | - | - | - | - | 25.42 | 661.2476 |
| 3,5,6,7,8,3',4'-Heptamethoxyflavone | + | + | + | + | + | 26.97 | 433.1488 |
| 4-hydroxybenzoic acid | + | + | + | - | - | 1.58 | 139.0386 |
| 4-hydroxybenzoic acid isomer (a) | - | - | + | - | - | 1.42 | 139.0386 |
| 5-methyl furan aldehyde | - | + | + | + | + | 1.95 | 111.0438 |
| a-Linolenic acid or its isomer (a) | +/+/+ | +/+/+ | +/+/+ | +/+/+ | +/+/+ | 26.63/28.36/30.92 | 279.2308 |
| Angeloylgomisin H or its isomer (a) | +/+ | +/+ | +/+ | +/+ | +/+ | 27.89/28.14 | 501.2473 |
| Anhydroicaritin or its isomer (a) | -/+/+/+ | -/+/+/+ | /+/-/-/+ | /+/+/+/+ | -/+/+/- | 23.58/25.15/25.38/26.02 | 369.1328 |
| Anhydroicaritin-3-O-Rhamnosyl-7-O -Glucopyranoside/Sagittatoside A | + | - | - | - | - | 25.15 | 677.2426 |
| Apigenin-7-O-gluA | - | + | - | - | - | 17.47 | 447.0931 |
| Apigenin | + | + | + | + | + | 24.59 | 271.0593 |
| Arbutin | + | + | + | + | + | 31.22 | 273.0958 |
| Baohuosu | + | + | + | + | + | 29.50 | 399.1425 |
| Benzoylpaeoniflorin/Paeonivayin or their isomer (b) | + | + | + | + | + | 25.21 | 585.1951 |
| Bergenin | + | + | + | + | + | 11.23 | 329.0880 |
| Calycosin-7-O-glu or its isomer (b) | + | + | + | + | + | 23.72 | 447.1287 |
| Calycosin | + | + | + | + | + | 22.51 | 285.0748 |
| Caryophyllene oxide or its isomer (a) | - | + | - | - | - | 24.73 | 221.1896 |
| Chamigrenal isomer or its isomer (a) | +/+/+ | +/+/+ | +/+/+ | +/+/+ | +/+/+ | 24.83/28.09/29.06 | 219.1737 |
| Chrysoeriol | - | + | + | + | - | 24.81 | 301.0698 |
| Corosolic acid | + | + | + | + | + | 28.56 | 473.3614 |
| Desmethylanhydroicaritin or its isomer (a) | +/+ | +/+ | +/+ | +/+ | +/+ | 24.37/25.01 | 355.1197 |
| Dihydroxy-trimethoxyflavone | - | + | + | + | - | 16.81 | 345.0985 |
| D-ribo-Phytosphingosine | + | - | - | + | - | 26.42 | 318.2998 |
| Ebeiedinone/Delavinone/Zhebeirine(puqiedinone) (a) | +/+ | +/+ | +/+ | +/+ | +/+ | 24.41/24.69 | 414.3354 |
| Epimedin A (Hexandraside F) | + | - | - | - | - | 22.31 | 839.2944 |
| Epimedin B | + | + | + | + | + | 22.98 | 809.2841 |
| Epimedin C (Baohuside VI) | + | + | - | - | - | 23.28 | 823.2999 |
| Epimedoside C | + | - | + | + | + | 24.23 | 517.1690 |
| Ethyl 4- hydroxybenzoate | + | + | + | + | - | 21.17 | 167.0700 |
| Formononetin | + | + | + | + | - | 25.34 | 269.0806 |
| Gomisin K1 | + | + | + | + | + | 28.52 | 403.2105 |
| Gomisin L1 or its isomer (a) | +/+/+/+ | +/+/+/+ | +/+/+/+ | +/+/+/+ | +/+/+/+ | 26.26/29.00/29.30/29.71 | 387.1800 |
| Hesperetin | - | + | + | + | + | 18.30 | 303.0875 |
| Hesperetin-7-O-glu or its isomer (a) | +/+ | +/- | -/- | -/- | -/- | 16.81/18.30 | 465.1396 |
| Hesperidin | + | + | + | - | + | 16.79 | 611.1998 |
| Hexamethoxyflavone (a) | + | + | + | + | + | 25.86 | 403.1381 |
| Icariin | + | - | + | - | + | 23.60 | 677.2423 |
| Icariside II (Baohuside I) | + | + | + | + | + | 26.02 | 515.1907 |
| Isopeimine A | + | + | + | + | + | 23.52 | 432.3473 |
| Isosalicin | + | + | + | - | + | 1.75 | 287.1112 |
| Isosinensetin | + | + | + | + | + | 25.66 | 373.1275 |
| Jasmonane | + | + | + | + | + | 26.24 | 165.1273 |
| Melitidin | + | - | - | - | - | 24.21 | 725.2269 |
| Meranzin/Isomeramazin (a) | +/+ | +/+ | +/+ | +/+ | +/+ | 18.00/25.56 | 261.1117 |
| Methyl eugeno | + | + | + | + | + | 26.26 | 179.1067 |
| Naringenin | + | - | + | + | + | 24.51 | 273.0750 |
| N-E-feruloyl tyramine | + | + | - | - | - | 22.20 | 314.1377 |
| Neoicariin/Wushanicariin/Icariside I or their isomer (b) | +/+/+ | -/-/+ | -/-/+ | -/-/+ | -/-/+ | 22.98/23.26/25.72 | 531.1852 |
| Neoisostegane | + | + | + | + | + | 29.00 | 415.1740 |
| Nobiletin | + | + | + | + | + | 26.61 | 403.1384 |
| Paeonol isomer | - | + | + | + | - | 21.80 | 167.0700 |
| Paeonol | + | + | + | + | + | 24.37 | 167.0698 |
| Peimine A | + | + | + | + | + | 19.17 | 432.3473 |
| Peiminine B isomer | + | + | + | + | + | 23.99 | 430.3306 |
| Peiminine B | + | + | + | + | + | 21.32 | 430.3306 |
| Peimisine isomer (a) | -/+/+/+/+ | -/+/+/+/+ | -/+/+/+/+ | +/+/+/+/+ | +/+/+/+/+ | 17.31/18.52/19.91/20.23/23.34 | 428.3171 |
| Peimisine | + | + | + | + | + | 17.72 | 428.3171 |
| Perillaldehyde | - | - | + | - | - | 24.35 | 151.1114 |
| Phenylalanine isomer | + | + | + | + | + | 1.95 | 166.0860 |
| p-Hydroxy-cinnamic acid | + | + | + | + | + | 1.97 | 165.0543 |
| Poncirin/Didymin | + | + | - | + | - | 22.42 | 595.2002 |
| Pyroglutamic acid | + | + | + | + | + | 2.22 | 130.0499 |
| Sagittatoside B | + | + | + | + | - | 25.38 | 647.2322 |
| Schisandrin A | + | + | + | + | + | 30.27 | 417.2258 |
| Schisandrin B(γ-Schisandrin) isomer (a) | +/+ | +/+ | +/+ | +/+ | +/+ | 27.93/28.12 | 401.1945 |
| Schisandrin B | + | + | + | + | + | 31.10 | 401.1945 |
| Schisandrin C isomer | - | - | + | + | + | 28.46 | 385.1631 |
| Schisandrin C | - | - | + | + | - | 31.75 | 385.1631 |
| Schisandrol B/Epigomisin O | - | + | + | + | + | 27.29 | 417.1900 |
| Schisanhenol | + | + | + | + | + | 29.00 | 403.2105 |
| Schisantherin A | + | + | + | + | + | 29.04 | 537.2109 |
| Schisantherin B | + | + | + | + | + | 28.95 | 515.2266 |
| Schisantherin C (Angeloylgomisin P) or its isomer (a) | -/+/+ | +/+/+ | +/+/+ | +/+/+ | +/+/+ | 27.39/29.14/29.63 | 515.2258 |
| Sinensetin | + | + | + | + | + | 26.16 | 373.1279 |
| Soyasaponin I | + | + | + | + | - | 25.23 | 943.5243 |
| Spathulenol | - | + | - | - | - | 24.51 | 221.1896 |
| Tangeretin | + | - | - | + | - | 27.29 | 373.1277 |

Table S3 Calibration curves, Linear ranges and LLOQs of the Bufei Yishen Formula compounds in serum

| Samples | Calibration curves | Correlation coeffificients (r) | | Linear ranges (ng/mL) | LLOQs (ng/mL) |
| --- | --- | --- | --- | --- | --- |
| Apigenin | y = 0.0653x + 0.0003 | | 0.9983 | 0.02-1000 | 0.02 |
| Calycosin | y = 0.1914x + 0.0026 | | 0.9951 | 0.02-1000 | 0.02 |
| Corosolic acid | y = 0.0116x – 0.0001 | | 0.9964 | 0.02-1000 | 0.02 |
| Epimedin B | y = 0.2607x + 0.0014 | | 0.9981 | 0.0172-860 | 0.0172 |
| Isosinensetin | y = 1.6714x + 0.0017 | | 0.9986 | 0.0124-620 | 0.0124 |
| Peimine A | y = 1.4571x + 0.0208 | | 0.9979 | 0.0246-615 | 0.0246 |
| Peiminine B | y = 1.4604x + 0.0257 | | 0.9924 | 0.0216-1080 | 0.0216 |
| Peimisine | y = 0.1696x + 0.0004 | | 0.998 | 0.0204-510 | 0.0204 |
| Schisandrin A | y = 0.258x + 0.0002 | | 0.9977 | 0.02-500 | 0.02 |
| Schisandrin B | y = 0.1065x - 0.0005 | | 0.9979 | 1.67-626.25 | 1.67 |
| Schisantherin A | y = 0.1837x + 0.0005 | | 0.9993 | 0.02-500 | 0.02 |
| Schisantherin B | y = 0.5542x - 0.0025 | | 0.9928 | 0.02-500 | 0.02 |
| Nobiletin | y = 1.4621x + 0.0055 | | 0.9998 | 0.0236-590 | 0.0236 |

Figure S1 The MS/MS spectra of the reference standard of Quinic acid

Figure S2 The MS/MS spectra of the reference standard of Pyroglutamic acid

Figure S3 The MS/MS spectra of the reference standard of Oxypaeoniflorin

Figure S4 The MS/MS spectra of the reference standard of Loganin

Figure S5 The MS/MS spectra of the reference standard of Rhoifolin

Figure S6 The MS/MS spectra of the reference standard of Hesperidin

Figure S7 The MS/MS spectra of the reference standard of Rosmarinic acid

Figure S8 The MS/MS spectra of the reference standard of Diosmin

Figure S9 The MS/MS spectra of the reference standard of Peimisine

Figure S10 The MS/MS spectra of the reference standard of Peimine A

Figure S11 The MS/MS spectra of the reference standard of Peiminine B

Figure S12 The MS/MS spectra of the reference standard of Epimedin A (Hexandraside F)

Figure S13 The MS/MS spectra of the reference standard of Calycosin

Figure S14 The MS/MS spectra of the reference standard of Epimedin B

Figure S15 The MS/MS spectra of the reference standard of Epimedin C (Baohuside VI)

Figure S16 The MS/MS spectra of the reference standard of Icariin

Figure S17 The MS/MS spectra of the reference standard of Ginsenoside Re

Figure S18 The MS/MS spectra of the reference standard of Ginsenoside Rb1

Figure S19 The MS/MS spectra of the reference standard of Perillaldehyde

Figure S20 The MS/MS spectra of the reference standard of Astragaloside Iv

Figure S21 The MS/MS spectra of the reference standard of Naringenin

Figure S22 The MS/MS spectra of the reference standard of Apigenin

Figure S23 The MS/MS spectra of the reference standard of Formononetin

Figure S24 The MS/MS spectra of the reference standard of Ginsenoside Rg2

Figure S25 The MS/MS spectra of the reference standard of Isosinensetin

Figure S26 The MS/MS spectra of the reference standard of Methyl eugenol

Figure S27 The MS/MS spectra of the reference standard of D-ribo-Phytosphingosine

Figure S28 The MS/MS spectra of the reference standard of Schisandrol A

Figure S29 The MS/MS spectra of the reference standard of Nobiletin

Figure S30 The MS/MS spectra of the reference standard of Tangeretin

Figure S31 The MS/MS spectra of the reference standard of Corosolic acid

Figure S32 The MS/MS spectra of the reference standard of Schisantherin B

Figure S33 The MS/MS spectra of the reference standard of Schisantherin A

Figure S34 The MS/MS spectra of the reference standard of Schisandrin A

Figure S35 The MS/MS spectra of the reference standard of Schisandrin B

Figure S36 The MS/MS spectra of the reference standard of Arbutin

Figure S37 The MS/MS spectra of the reference standard of Schisandrin C

Figure S38 A total ion chromatogram of the mix rat serum after administrated of BYF extract at1 h.

Figure S39 The content of Bufei Yishen Formula components in the rat serum
